# Anomalous spin precession systematic effects in the search for a muon EDM using the frozen-spin technique

**DOI:** 10.1140/epjc/s10052-024-12604-0

**Published:** 2024-03-12

**Authors:** G. Cavoto, R. Chakraborty, A. Doinaki, C. Dutsov, M. Giovannozzi, T. Hume, K. Kirch, K. Michielsen, L. Morvaj, A. Papa, F. Renga, M. Sakurai, P. Schmidt-Wellenburg

**Affiliations:** 1 Paul Scherrer Institut, 5232 Villigen PSI, Switzerland; 2 CERN Beams Department, Esplanade des Particules 1, 1211 Meyrin, Switzerland; 3 Istituto Nazionale di Fisica Nucleare, Sez. di Roma, P.le A. Moro 2, 00185 Rome, Italy; 4 Istituto Nazionale di Fisica Nucleare, Sez. di Pisa, Largo B. Pontecorvo 3, 56127 Pisa, Italy; 5 ETH Zürich, 8092 Zurich, Switzerland; 6 École Polytechnique, Route de Saclay, 91128 Palaiseau Cedex, France; 7 Present Address: University College London, Gower Street, London, WC1E 6BT UK

## Abstract

At the Paul Scherrer Institut (PSI), we are developing a high-precision apparatus with the aim of searching for the muon electric dipole moment (EDM) with unprecedented sensitivity. The underpinning principle of this experiment is the frozen-spin technique, a method that suppresses the spin precession due to the anomalous magnetic moment, thereby enhancing the signal-to-noise ratio for EDM signals. This increased sensitivity enables measurements that would be difficult to achieve with conventional $g - 2$ muon storage rings. Given the availability of the ${125}\,{\textrm{MeV}/\textit{c}}$ muon beam at PSI, the anticipated statistical sensitivity for the EDM after a year of data collection is ${6\times 10^{-23}}\,{e\!\cdot \!\textrm{cm}}.$ To achieve this goal, it is imperative to do a detailed analysis of any potential spurious effects that could mimic EDM signals. In this study, we present a quantitative methodology to evaluate the systematic effects that might arise in the context of the frozen-spin technique utilised within a compact storage ring. Our approach involves the analytical derivation of equations governing the motion of the muon spin in the electromagnetic (EM) fields intrinsic to the experimental setup, validated through numerical simulations. We also illustrate a method to calculate the cumulative geometric (Berry’s) phase. This work complements ongoing experimental efforts to detect a muon EDM at PSI and contributes to a broader understanding of spin-precession systematic effects.

## Introduction

The existence of a permanent EDM in any elementary particle suggests a violation of Charge-Parity (CP) symmetry. Within the framework of the Standard Model (SM) of particle physics, EDMs are predicted to be remarkably small, despite the substantial CP-violating phase provided by the Cabibbo–Kobayashi–Maskawa matrix. In fact, they are so small that they are beyond the reach of any imminent measurements. Nevertheless, numerous SM extensions allow for substantial CP violating phases, which can result in large EDMs [1, 2]. Recently, the EDM of the muon has drawn significant attention, due to a persistent tension between the experimental results for the muon anomalous magnetic moment (AMM) [3, 4] and the theoretical SM predictions [5].

Farley et al. [6–8] proposed a method to measure EDMs in storage rings, known as the frozen-spin technique. The frozen-spin technique cancels the anomalous $(g-2)$ precession by applying a radial electric field perpendicular to the momentum of the stored particles and to the magnetic field, so that any remaining precession is a consequence of the EDM. In a real-world storage ring, where precession due to the AMM cannot be completely suppressed, EDM-like signals may be induced. Such systematic effects can reduce experimental sensitivity or result in a signal mimicking a genuine EDM.

A non-zero EDM manifests itself through a precession of the spin around the electric-field vector in the particle’s frame of reference. In the case of muons, the spin precession can be measured by studying the direction of the emitted decay positrons, which is correlated to the spin direction. Our study is focused on the systematic effects induced by coupling of the magnetic dipole moment to the EM field of the experiment. We delve into both the dynamic and geometric phases of the spin for muons circulating within the confines of a compact storage ring that employs the frozen-spin technique. Although we strive to maintain sufficient generality in our derivations to ensure their applicability across different scenarios, our discussions are rooted in the context of the ongoing experimental efforts. Specifically, we have evaluated potential systematic effects associated with the ongoing effort to search for a muon EDM at PSI.

The main part of this paper is separated in four sections. First we present the details of the experimental setup which will be used to search for the EDM of the muon. The frozen-spin technique is elaborated upon and the expected statistical sensitivity is given. In the next section we derive analytical expressions for the motion of the muon spin in an idealised version of the EM fields of the experiment. The results are verified by comparison with simulations using Geant4 [9–11], shown in detail in the appendix. The derivations treat both the dynamic phase build-up, as well as the potential for generation of geometric phases. Next we consider effects arising from possible deviations of the real EM field from the nominal one. In this part we also derive effects arising from deviations of the initial spin and momentum of the muon at the start of a measurement. Finally, in the discussion, we use the derived analytical equations to calculate limits on parameters of the experimental setup such that any possible systematic effect is lower than our target sensitivity for the search for a muon EDM.

## Search for the muon EDM at PSI

The search for a muon EDM at PSI will rely on a storage ring inside a compact solenoid with inner diameter less than a meter [12, 13]. The anti-muons will be injected into the solenoid one by one, through a superconducting injection channel [14] and subsequently kicked by a pulsed magnetic field into a stable orbit within a weakly-focusing field [15]. Two concentric cylindrical electrodes will provide a radial electric field at the position of the muon orbit. The strength of this electric field must be precisely tuned so as to satisfy the frozen-spin condition, where the anomalous spin precession is cancelled and the spin remains aligned with the muon momentum for the duration of its lifetime.

**Fig. 1:**
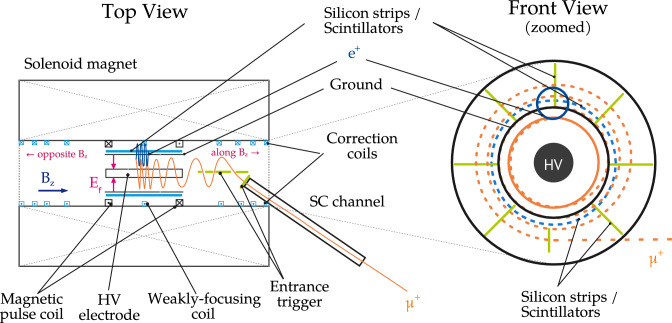
Illustration of the Phase I muon EDM experimental device employing a compact storage ring inside a solenoid magnet

As the muon decays, the direction of its spin can be statistically inferred from the trajectory of the emitted decay positron. The parity violation in the weak decay results in a preference for high-energy positrons to be emitted in the direction of the muon spin. The EDM will be calculated based on the change in asymmetry, *dA*/*dt*,  where $A(t) = (N_\uparrow (t) - N_\downarrow (t))/(N_\uparrow (t) + N_\downarrow (t)),$ which measures the difference between the number of positrons emitted along or opposite the main magnetic field, see Fig. 1. Detectors positioned symmetrically on both sides of the plane defined by the ideal muon orbit will monitor the direction of emission.

A staged approach has been adopted for this project. The initial phase (Phase I) will focus on demonstrating the feasibility of all critical techniques, with the goal of achieving a sensitivity to the muon EDM $d_\mu$ better than $\sigma (d_\mu )\le {3\times 10^{-21}}\,{e\!\cdot \!\textrm{cm}}.$ The next phase (Phase II) aims to achieve a sensitivity of better than ${6\times 10^{-23}}\,{e\!\cdot \!\textrm{cm}},$ which would represent an improvement of more than three orders of magnitude over the current experimental limit of $d_\mu \le {1.8\times 10^{-19}}\,{e\!\cdot \!\textrm{cm}}$ (95% CL) [16].

To reach this target sensitivity, it is crucial to ensure that potential systematic effects leading to a false EDM signal are adequately controlled. In particular, we investigate the impact of EM field irregularities on the experimental results. For this, we study the relativistic spin motion of a positively charged $(+e)$ muon of mass *m* with momentum $\vec {p}$ in electric $\vec {E}$ and magnetic $\vec {B}$ fields described by the Thomas-BMT equation [17–19], with an additional term describing the effect of the EDM on the spin precession rate, namely

1 $$\begin{aligned} \vec {\varOmega }= & {} \vec {\varOmega }^{\mathchoice{}{}{\scriptscriptstyle }{}\text {AMM}}+ \vec {\varOmega }^{\mathchoice{}{}{\scriptscriptstyle }{}\text {EDM}}\nonumber \\= & {} -\frac{e}{m}\left[ a\vec {B}-\frac{a\gamma }{\left( \gamma +1\right) }\left( \vec {\beta }\cdot \vec {B}\right) \vec {\beta }\right. \nonumber \\{} & {} \left. -\left( a+\frac{1}{1-\gamma ^2}\right) \frac{\vec {\beta }\times \vec {E}}{c}\right] \nonumber \\{} & {} -\frac{\eta e}{2m}\left[ \vec {\beta }\times \vec {B}+\frac{\vec {E}}{c}-\frac{\gamma }{c(\gamma +1)}\left( \vec {\beta }\cdot \vec {E}\right) \vec {\beta }\right] , \end{aligned}$$

where $\vec {\beta }=\vec {p}c/E$ and $\gamma =\left( 1-\beta ^2\right) ^{-1/2}$ are the relativistic factors with total energy *E*,  *a* is the anomalous magnetic moment, and $\eta =4d_\mu m c/(e\hbar )$ the gyro-electric ratio multiplied by 2*mc*/*e* which is the dimensionless constant describing the size of the EDM.

The second line of Eq. (1) represents the anomalous precession frequency $\vec {\varOmega }^{\mathchoice{}{}{\scriptscriptstyle }{}\text {AMM}},$ the difference between the Larmor precession and the cyclotron precession, due to the AMM. The last line represents the precession $\vec {\varOmega }^{{\mathchoice{}{}{\scriptscriptstyle }{}\text {EDM}}}$ due to the EDM coupling to the electric field in the boosted reference frame of the moving muon.

The experimental setup proposed for the search for a muon EDM is based on the ideas and concepts discussed in [6, 12, 13, 15]. The salient feature of the proposed search is the cancellation of the precession due to the anomalous magnetic moment by meticulously choosing a radial electric field, and thus fully exploiting the large electric field $\gamma c\vec {\beta }\times \vec {B}\approx {1}\,{\mathrm{GV/m}}$ in the rest frame of the muon to achieve a perpendicular precession $(\vec {\varOmega } \, \bot \, \vec {B})$ only. By examining Eq. (1), we can counteract the anomalous precession term by applying an electric field such that:

2 $$\begin{aligned} a\vec {B} = \left( a+\frac{1}{1-\gamma ^2}\right) \frac{\vec {\beta }\times \vec {E_{\textrm{f}}}}{c}. \end{aligned}$$

In the case of $\vec {\beta }\cdot \vec {B}=\vec {\beta }\cdot \vec {E}=0,$ $\vec {B}\cdot \vec {E}=0,$ and assuming $a \ll 1/(1-\gamma ^2),$ which is a good approximation for small $\gamma ,$ we find a required field strength of $\vert E_{\textrm{f}} \vert \approx a c\beta \gamma ^2 \vert B \vert ,$ which is approximately ${0.3}\,{\mathrm{MV/m}}$ for the Phase I experiment. Hence, by selecting the exact field condition of Eq. (2), the cyclotron precession frequency is modified such that the relative angle between the momentum vector and the spin remains unchanged if $\eta =0;$ the spin is “frozen”.

### Sensitivity to the muon EDM

Using Eq. (1) and assuming that $\vec {\beta } \cdot \vec {E} = 0,$ $\vec {\beta } \cdot \vec {B} = 0$ and $|E |\ll c|\vec {\beta }\times \vec {B}|$ (as evident from the aforementioned ${0.3}\,{\mathrm{MV/m}}\ll {1}\,{\mathrm{GV/m}}),$ the spin precession angular velocity due to a non-zero EDM is:

3 $$\begin{aligned} \vec {\varOmega }^{\mathchoice{}{}{\scriptscriptstyle }{}\text {EDM}}= \frac{\eta }{2}\frac{e}{m}\vec {\beta } \times \vec {B}, \end{aligned}$$

**Fig. 2:**
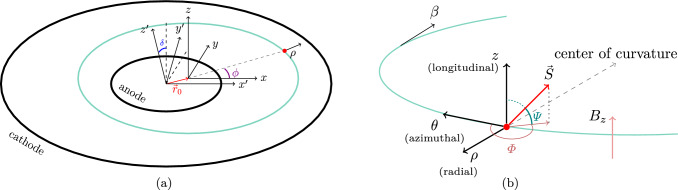
Representations of the reference frames used throughout the paper. **a** Cartesian reference frame used to describe the *E*-field in the particle rest frame. The origin of the (*x*, *y*, *z*) reference frame coincides with the centre of a given muon orbit, where the *z* axis is parallel to the field of the main solenoid and the *xy* plane lies in the orbit plane. The origin of the primed reference frame is at the centre of the cylindrical electrodes, where $z'$ runs parallel to the central axes of the inner and outer electrodes (anode and cathode). The angle $\delta$ is the angle between the central axes of the electrodes and the main solenoid. **b** The curvilinear (Frenet–Serret) reference coordinate system used to derive the motion of the spin in the EM fields of the experiment. The axis $\theta$ follows the momentum of the muon and *z* is always parallel to the main solenoid magnetic field $B_z.$ The vector $\vec {S}$ is the normalised particle spin. The angle $\varPhi$ is the azimuthal spin phase (in the plane of the orbit), and $\varPsi$ is the complementary angle to the polar angle

Note that the coordinate system used here and throughout this work is such that it follows the reference particle orbit (similar to [20, 21]) as sketched in Fig. 2b. The initial orientation of the spin $\vec {S} = (S_\theta , S_\rho , S_z)$ in spherical coordinates is

4 $$\begin{aligned} \varPhi _0 = \arctan \left( \frac{S_\rho }{S_\theta } \right) , \quad \varPsi _0 = \frac{\pi }{2} - \arccos \left( S_z \right) , \end{aligned}$$

where $|\vec {S} \vert = 1,$ $\varPhi$ is the azimuthal spin phase, i.e., in the plane of the orbit, and $\varPsi$ is the complementary angle to the polar angle.

From the assumption that the *E*-field and the *B*-field are perpendicular to each other and to the muon velocity, the only sizeable component of $\vec {\varOmega }_{\mathchoice{}{}{\scriptscriptstyle }{}\text {EDM}}$ is the radial component,

5 $$\begin{aligned} \varOmega ^{\mathchoice{}{}{\scriptscriptstyle }{}\text {EDM}}_\rho = {\dot{\varPsi }} = \frac{2c}{\hslash }\beta _\theta B_z d_\mu , \end{aligned}$$

where we have replaced $\eta$ with the expression for $d_\mu .$

In Phase I and Phase II we will store muons with $\beta _\theta = 0.256$ $(p = {28}\,{\textrm{MeV}/\textit{c}})$ and 0.764 $(p = {125}\,{\textrm{MeV}/\textit{c}}),$ respectively, in a magnetic field of strength $B_z={3}\,{\textrm{T}}.$ This results in angular velocities for an EDM equal to the annual statistical sensitivity of the muon EDM measurement [22] of:

6 $$\begin{aligned} {\dot{\varPsi }}_{\textrm{I}}&= {21.15}\text { rad/s} \quad \text {for}\ d_\mu = {3\times 10^{-21}}\,{e\!\cdot \!\textrm{cm}}, \end{aligned}$$

7 $$\begin{aligned} {\dot{\varPsi }}_{\textrm{II}}&= {1.26}\text { rad/s} \quad \text {for}\ d_\mu = {6\times 10^{-23}}\,{e\!\cdot \!\textrm{cm}}. \end{aligned}$$

The radius of the orbit is $\rho _0 = {31}\,{\textrm{mm}}$ for the first phase and ${134}\,{\textrm{mm}}$ for the second. The required frozen spin field is $E_{\textrm{f}} = {287}\,{\mathrm{kV/m}}$ and $\textrm{E}_{\textrm{f}} = {1.92}\,{\mathrm{MV/m}}$ for Phase I and II respectively.

## The spin motion of muons in the experiment

As a starting point to the analysis of possible systematic effects we derive an approximate analytical expression for the spin motion in the field configuration characteristic of the frozen-spin technique. For this purpose we approximate the magnetic field of the solenoid in the region of the storage ring as a uniform magnetic field oriented along the *z*-axis; the weakly-focusing field by the first-order approximation of a field generated by a circular coil; and, the electric field as a radial field produced by the potential difference between two infinite coaxial cylindrical electrodes. We then parameterise the most important and most likely imperfections of these fields and estimate their effect on the spin precession in the following sections.

### Spin precession around the radial axis

The longitudinal position of a particle with charge *e*,  mass *m*,  and velocity $c\vec {\beta }$ is given by the solution of:

8 $$\begin{aligned} \ddot{z} = \frac{e}{\gamma m} \left( E_z + c \beta _\theta B_\rho (z) + c\beta _\rho B_\theta (z) \right) , \end{aligned}$$

where $B_\rho (z) \approx z \frac{\partial B_\rho (\rho _0)}{\partial z} = z \partial _z B_\rho (\rho _0),$ and we assume that a constant non-zero *z*-component of the electric field exists. In general, the last term $\beta _\rho B_\theta (z) \ll \beta _\theta B_\rho (z)$ as $\beta _\rho$ is practically zero for stored particles and $B_\theta$ is zero if there is no electrical current flowing through the area enclosed by the orbit. Therefore, the term is ignored in the further discussion and the solution of the differential equation becomes that of a harmonic oscillator with longitudinal displacement

9 $$\begin{aligned} z(t) = z_0\cos (\omega _{\textrm{b}} t + \varphi _0) + \frac{1}{\partial _z B_\rho }\frac{E_z}{c\beta _\theta }, \end{aligned}$$

where $\omega _{\textrm{b}}$ is the angular velocity of the longitudinal betatron oscillation, and $z_0$ is the amplitude of the longitudinal displacement. It can be expressed in terms of the field gradient index $n = \frac{\rho _0}{B_0}\partial _z B_\rho$ as $\omega _{\textrm{b}} = \omega _{\textrm{c}} \sqrt{n},$ where $\omega _{\textrm{c}}= -eB_0/\gamma m$ is the cyclotron angular velocity, $\rho _0$ is the radius of the nominal orbit, and $B_0$ is the magnetic field of the main solenoid. The particles in the storage ring also experience a horizontal betatron oscillation (in the plane of the orbit), with angular velocity $\omega _{\textrm{h}} = \omega _{\textrm{c}} \sqrt{1 - n},$ that corresponds to oscillations in $B_z$ and does not directly lead to spin precession mimicking the EDM signal. In a compact storage ring configuration $\omega _{\textrm{h}} \approx \omega _{\textrm{c}}$ since $n \ll 1,$ such that a small difference between these two frequencies leads to a slow precession of the muon orbit whose effects are explored in Sect. 4.2.

The relative precession of the spin due to the coupling of the AMM to the radial magnetic field of the weakly-focusing field is

10 $$\begin{aligned} \varOmega ^{\mathchoice{}{}{\scriptscriptstyle }{}\text {WF}}_\rho= & {} -\frac{ea}{m}B_\rho (z(t)) \nonumber \\\approx & {} -\frac{ea}{m}\left[ \partial _z B_\rho z_0 \cos (\omega _{\textrm{b}} t + \varphi _0) - \frac{1}{c\beta _\theta } E_z\right] , \end{aligned}$$

where the index $\rho$ denotes the radial component of $\vec {\varOmega }.$

Another source of radial precession that has to be considered is the radial magnetic field in the reference frame of the muon due to a non-zero longitudinal electric field in the laboratory reference frame. For a non-zero longitudinal electric field, $|E_z|>0,$ we obtain

11 $$\begin{aligned} \varOmega ^{\scriptscriptstyle {}E_z}_\rho = -\frac{e}{mc} \left( a - \frac{1}{\gamma ^2 -1} \right) \beta _\theta E_z, \end{aligned}$$

for the radial component only, by applying the T-BMT equation.

Further, a radial spin precession could also be caused by a radial *B*-field $B^{\mathchoice{}{}{\scriptscriptstyle }{}\textrm{K}}_\rho$ due to residual currents in coils or eddy currents induced by the short, $\varDelta t_{\textrm{pulse}}\approx {100}\,{\textrm{ns}},$ magnetic pulse used to kick muons onto a stable orbit, see Fig. 1. This field can be described by a superposition of periodic oscillations,

12 $$\begin{aligned} B^{{\mathchoice{}{}{\scriptscriptstyle }{}\textrm{K}}}_\rho (t) = \int _0^\infty A_\rho (\omega ) \cos (\omega t + b_0(\omega )) d\omega , \end{aligned}$$

where $A_\rho (\omega )$ is the oscillation amplitude as a function of the angular frequency $\omega$ and $b_0(\omega )$ is an arbitrary frequency-dependent phase. Possible systematic effects due to such oscillations are explored in detail in Sect. 4.1.

Combining Eqs. (10) and (11), and including the term for an arbitrary radial magnetic field $B^{\mathchoice{}{}{\scriptscriptstyle }{}\textrm{K}}_\rho (t)$ as in Eq. (12), one obtains the total angular velocity of the radial precession due to the AMM around the $\rho$-axis

13 $$\begin{aligned} \varOmega ^{\mathchoice{}{}{\scriptscriptstyle }{}\text {AMM}}_\rho= & {} -\frac{ea}{m} \left[ \frac{1}{c}\left( 1-\frac{1}{a(\gamma ^2 - 1)} - \frac{1}{\beta ^2_\theta }\right) \beta _\theta E_z \right. \nonumber \\{} & {} +\partial _z B_\rho z_0 \cos (\omega _{\textrm{b}} t + \varPhi _0) + B^{\mathchoice{}{}{\scriptscriptstyle }{}\textrm{K}}_\rho (t)\bigg ]. \end{aligned}$$

We are interested in the average angular velocity over many muon orbits. In this case, the average due to the betatron oscillations is zero, as $\langle \cos (\omega _{\textrm{b}} t) \rangle = 0$ for $t \gg \omega _{\textrm{b}}^{-1}.$ In general, the azimuthal velocity $\beta _\theta$ and the longitudinal electric field $E_z$ are not correlated, thus the average over time of their product is the product of their averages

14 $$\begin{aligned} \left\langle \varOmega ^{{\mathchoice{}{}{\scriptscriptstyle }{}\text {AMM}}}_\rho \right\rangle= & {} -\frac{ea}{mc} \left\langle \left( 1 - \frac{1}{a(\gamma ^2 - 1)} - \frac{1}{\beta _\theta ^2}\right) \beta _\theta \right\rangle \langle E_z \rangle \nonumber \\{} & {} - \frac{ea}{m}\left\langle B^{\mathchoice{}{}{\scriptscriptstyle }{}\textrm{K}}_\rho (t) \right\rangle . \end{aligned}$$

Note that here $E_z$ is a static uniform field and $B^{\mathchoice{}{}{\scriptscriptstyle }{}\textrm{K}}_\rho$ is an arbitrary time-dependent field. Effects of their time stability and spatial uniformity are discussed in Sect. 4.1.

Although the average of the betatron oscillations is zero, oscillations can still occur around two perpendicular axes, potentially leading to the accumulation of a geometrical, also known as Berry’s, phase [23]. Additionally, potential systematic effects may arise from the approximation $t \gg \omega _{\textrm{b}}^{-1}.$ These sources of systematic effects are explored in more detail in Sect. 3.5.

### Azimuthal spin precession

When the muons circulate in the storage ring, they oscillate longitudinally (along the *z*-axis) around an equilibrium orbit (betatron oscillation). The equilibrium orbit is perpendicular to the longitudinal magnetic field. In the absence of other fields the betatron oscillation results from the weakly-focusing field. Due to this betatron motion, the momentum vector of the particle is not at all times perpendicular to the longitudinal magnetic field, leading to a non-zero projection of the magnetic field along its trajectory. This component of the field is proportional to the angle, $\zeta = \pi /2 - \angle (\vec {\beta }, \vec {B}),$ between the muon momentum and the plane of the equilibrium orbit. In turn, $\tan \zeta = p_z / p_\theta \approx \sin \zeta$ and $p_z$ oscillates as $p_z = p_{z_0} \sin (\omega _{\textrm{b}} t).$ The muon is therefore exposed to an oscillating azimuthal field,

15 $$\begin{aligned} B_\theta (t) = -B_z \sin \zeta \approx -B_z \frac{p_{z_0}}{p_\theta } \sin ( \omega _{\textrm{b}} t), \end{aligned}$$

where the momentum

16 $$\begin{aligned} p_{z_0} = e c \beta \partial _z B_\rho z_0 \int _0^{\frac{\pi }{2 \omega _{\textrm{b}}}} \cos (\omega _{\textrm{b}} t) dt = \frac{e c \beta z_0}{\omega _{\textrm{b}}} \partial _z B_\rho , \end{aligned}$$

is the *z*-component of the momentum at the nominal orbit plane (at $$z=0).$$ This also means that there will be a non-zero *z*-component of the velocity, given by

17 $$\begin{aligned} \beta _z(t) = \frac{p_{z_0}}{p_\theta }\,\beta _\theta \sin ( \omega _{\textrm{b}} t) . \end{aligned}$$

If the radial electric field $E_\rho$ is correctly set to the value $E_{\textrm{f}}$ required for the frozen-spin technique, then there will be no oscillations around the azimuthal $\theta$-axis as the electric field perfectly counteracts the precession induced by the coupling of the AMM to the longitudinal field of the solenoid. However, if $E_\rho \ne E_{\textrm{f}}$ there will be imperfect cancellation of the $g-2$ precession around $\theta$ that is proportional to the mean excess radial component $E_{\textrm{ex}} = E_\rho - E_{\textrm{f}}$ affecting the muon dynamics, namely

18 $$\begin{aligned} \varOmega ^{\scriptscriptstyle \varDelta E}_\theta = \frac{e}{m} \left( a - \frac{1}{\gamma ^2 - 1} \right) \frac{\beta _z(t)}{c}E_{\textrm{ex}}. \end{aligned}$$

In a realistic scenario where the orbit centre is displaced from the *E*-field central axis, the radial component $E_\rho$ would be position and momentum dependent. This is explored in the next Sect. 3.3.

Taking into account Eq. (15), we can approximate the angular velocity of the spin precession along the azimuthal $\theta$-axis to

19 $$\begin{aligned} \varOmega ^{\,\scriptscriptstyle \beta \cdot B}_\theta = \frac{e}{m} \left( \frac{a\gamma }{\gamma + 1}\right) \beta _\theta ^2 B_\theta (t), \end{aligned}$$

due to the term which is second-order with respect to the velocity $(\vec {\beta } \cdot \vec {B})\vec {\beta }$ in the Thomas-BMT equation, where $B_\theta (t)$ could have contributions from a residual magnetic field $B^{\mathchoice{}{}{\scriptscriptstyle }{}\textrm{K}}_\theta$ due to the magnetic kick or induced eddy currents, similar to the time-dependent radial field shown in Eq. (12).

In summary, we combine Eqs. (17), (18), and (19), and include an arbitrary azimuthal *B*-field, $B^{\mathchoice{}{}{\scriptscriptstyle }{}\textrm{K}}_\theta (t),$ for the total azimuthal angular velocity,

20 $$\begin{aligned} \varOmega ^{\mathchoice{}{}{\scriptscriptstyle }{}\text {AMM}}_\theta= & {} \frac{e}{m}\frac{p_{z_0}}{p_\theta } \sin ( \omega _{\textrm{b}} t) \left[ \left( a - \frac{1}{\gamma ^2 - 1} \right) \frac{\beta _\theta }{c}E_{\textrm{ex}} \right. \nonumber \\{} & {} - \left( \frac{a\gamma }{\gamma + 1}\right) \beta _\theta ^2 B_z\bigg ] - \frac{ea}{m} B^{\mathchoice{}{}{\scriptscriptstyle }{}\textrm{K}}_\theta (t), \end{aligned}$$

due to the AMM. Averaged over $t \gg \omega _{\textrm{b}}^{-1}$ results in

21 $$\begin{aligned} \left\langle \varOmega ^{\mathchoice{}{}{\scriptscriptstyle }{}\text {AMM}}_\theta \right\rangle = -\frac{e a}{m} \langle B^{\mathchoice{}{}{\scriptscriptstyle }{}\textrm{K}}_\theta (t) \rangle . \end{aligned}$$

On the closed orbit, the average azimuthal magnetic field $\langle B^{\mathchoice{}{}{\scriptscriptstyle }{}\textrm{K}}_\theta \rangle$ is equal to zero when no current flows through the area enclosed by the muon orbit. Despite the fact that $\left\langle \varOmega ^{\mathchoice{}{}{\scriptscriptstyle }{}\text {AMM}}_\theta \right\rangle =0,$ it remains informative to identify and quantify the primary sources of oscillations. Furthermore, when these oscillations are combined with those around the other two axes, it is possible that a geometric phase may accumulate, as discussed in Sect. 3.5.

### Description of the electric field in the storage-ring region

The radial electric field $E_\rho ,$ essential for the frozen-spin condition, may not be constant along the muon orbit. This might occur if the axes of the electrodes are not aligned with that of the solenoid field or if the muon orbit is not centred to the axes of the electrodes.

To determine the components of the electric field in the muon reference frame, we consider a purely radial electric field generated by perfectly coaxial cylindrical electrodes with radii *A* and *B* with $A<B.$ In the reference frame $(x', y', z')$ of the electrodes (see Fig. 2a),

22 $$\begin{aligned} \vec {E}'(x', y', z') = \frac{V}{\log {\frac{B}{A}}}\begin{pmatrix}\frac{x'}{(x')^2 + (y')^2} \\ \\ \frac{y'}{(x')^2 + (y')^2} \\ \\ 0\\ \end{pmatrix}, \end{aligned}$$

where $z'$ is parallel to the solenoid axis, which might be displaced with respect to the centre of the muon orbit. By applying a rotation, $R(\delta ),$ around the $y'$-axis by an angle $\delta ,$ we transform the electric field,

23 $$\begin{aligned} \vec {E} = R_z(\delta ) \vec {E}'(R_z^{-1}(\delta )\vec {r} + \vec {r}_0), \end{aligned}$$

in the rest frame of the muon, where $\vec {r}_0 = (x'_0, y'_0, 0)$ is the displacement between the centre of the muon orbit and the position of the electrode’s axis. Due to the cylindrical symmetry of the *E*-field around its central axis, we can always select the reference frame such that arbitrary displacements can be represented in this manner. Therefore, the electric field in the reference frame defined by the longitudinal magnetic field may be written as

24 $$\begin{aligned} \vec {E}(x, y, z) = \frac{V}{\log {\frac{B}{A}}} \begin{pmatrix} \frac{x}{r^2} \cos \delta \\ \\ \frac{y}{r^2} \\ \\ -\frac{x}{r^2} \sin \delta \end{pmatrix}, \end{aligned}$$

where $$y = y' + y'_0,$$ $$x = x'_0 + x' \cos \delta - y' \sin \delta ,$$ and $$r^2 = x^2 + y^2.$$

The average of the radial electric field over the circular orbit of the muon,

25 $$\begin{aligned} {\tilde{E}}_\rho = \langle E_\rho \rangle _\phi = \frac{1}{2 \pi } \int _0^{2\pi } E_\rho (\rho , \phi , z) d\phi , \end{aligned}$$

can be obtained most easily by representing $\vec {E}(\vec {r})$ in cylindrical coordinates and integrating over the angle $\phi ,$ shown in Fig. 2a. To consider the spin motion due to the cyclotron motion in the electric field, we approximate the radial component,

26 $$\begin{aligned} E_\rho (t) \approx {\tilde{E}}_\rho + \frac{1}{2}\left( E_{\rho ,{\textrm{max}}} - E_{\rho ,{\textrm{min}}}\right) \cos (\omega _{\textrm{c}} t + b_0), \end{aligned}$$

where $E_{\rho , {\textrm{max}}}$ and $E_{\rho ,{\textrm{min}}}$ are the maximal and minimal values of the electric field along a muon orbit and $b_0$ is the initial phase of the muon position along the orbit.

Note that Eq. (25) is valid only in the case of a circular orbit. In this case, it can be shown numerically that

27 $$\begin{aligned} \left\langle E(\rho , z) \right\rangle _\phi = \left\langle E'(\rho , z)\right\rangle _\phi , \end{aligned}$$

which means that a tilt of the concentric assembly of inner and outer electrodes with respect to the main *B*-field axis does not influence the average frozen-spin condition and, more importantly, does not change the net $E_z$ component. Another significant consequence of this finding is that displaced muon orbits would still experience the same average radial component as the nominal orbit, ensuring that displacements do not influence the storage of muons.

As the centripetal force due to the *B*-field is about a factor $10^3$ larger than that due to the *E*-field, and the expected misalignment between the centre of the orbit and the centre of the inner electrode is small, the circular orbit approximation holds well. Another source for non-circular orbits is a non-uniform magnetic field, $B_z,$ which is discussed in Sect. 4.1.

### EDM-like spin precession

The signature of an EDM is the time-dependent asymmetry between decay positrons emitted along or opposite the *B*-field (*z*-axis), which is proportional to the change in the projection of the spin along the *z*-axis. The angular velocity of Eqs. (13) and (20) projected along the *z*-axis is

28 $$\begin{aligned} \vec {\varOmega }^{\mathchoice{}{}{\scriptscriptstyle }{}\text {AMM}}\cdot {\hat{z}}= & {} \varOmega ^{\mathchoice{}{}{\scriptscriptstyle }{}\text {AMM}}_\rho \cos (\omega _z t + \varPhi _0) \nonumber \\{} & {} + \varOmega ^{\mathchoice{}{}{\scriptscriptstyle }{}\text {AMM}}_\theta \sin (\omega _z t + \varPhi _0), \end{aligned}$$

where

29 $$\begin{aligned} \omega _z = -\frac{e}{m}\left[ aB_z - \left( a + \frac{1}{1 - \gamma ^2} \right) \frac{\beta }{c}{\tilde{E}}_\rho \right] \end{aligned}$$

is the angular velocity of the precession around *z* due to an imperfect cancellation of the $g-2$ precession and ${\hat{z}}$ is the unit vector along *z*. Note that this equation holds for a two-dimensional motion and is only an approximation for three dimensions. A more thorough study of the three dimensional case can be found in [24]. In reality, $E_\rho$ will oscillate, according to Eq. (26), with frequency $\omega _{\textrm{c}},$ due to the changing distance between the muon and the centre of the electrodes generating the electric field. $B_z$ will oscillate with frequency $\omega _{\textrm{b}}$ due to the variation of the weakly-focusing field. In a well-tuned frozen-spin experiment, $\omega _z$ is much smaller than $\omega _{\textrm{b}}$ and $\omega _{\textrm{c}},$ and the rotation of the spin around *z* can be approximated with a constant angular velocity using the average values of $E_\rho$ and $B_z.$ The total longitudinal rotation of the spin with respect to the momentum is

30 $$\begin{aligned} \varPsi (t) = \int _0^t \vec {\varOmega }^{\mathchoice{}{}{\scriptscriptstyle }{}\text {AMM}}\left( t'\right) \cdot {\hat{z}}\,{\text {d}}t'. \end{aligned}$$

To verify the validity of the equation derived for the total longitudinal rotation corresponding to the EDM signal, we have set up a Geant4 Monte Carlo simulation of the storage ring region of the experimental setup. While Geant4 is not a usual choice for storage ring simulations it was deemed optimal for this experiment as the detectors, field generating elements (coils, electrodes), and muon orbit are in close proximity and very much interlinked. Though the available integration algorithms in Geant4 are non-symplectic, the effects on the tracked position, momentum, and spin direction are smaller than the effects from running the simulations with ${1\times 10^{-7}}\,{\textrm{mbar}}$ air pressure within the experimental volume. Additionally, testing the code with asymptotically small step sizes in the range of 0.01 mm to 2.0 mm converged to a stable solution for small step size on the nominal orbit, indicating that the direction of the muon spin as a function of time did not show significant variations with changes in step size within this range. In a future extension of this study we plan to verify the spin and beam dynamics analytical estimations by independent high-precision simulations.

The EM field in the simulation is read from fieldmaps generated by finite element simulations using the software ANSYS Maxwell3D [25]. The effects of finite spacing between points on a regular grid on which the EM field is defined have a significantly larger impact than the choice of integration scheme and step size. The optimisation of the EM field generation as well as the verification of the simulation and derivation of analytical equations are shown in detail in Appendix A.

### Spin precession due to geometric phases

The geometric phase, also known as Berry’s phase, is a phase difference acquired over the course of a cycle in parameter space when the system evolves adiabatically [23].^1^ Such cycles in the parameter space can occur due to the periodic oscillations of stored muons in the non-uniform electric and magnetic fields of the experimental device. In classical parallel transport, the phase accumulation is equal to the solid angle subtended by a vector on the spherical surface in parameter space. For quantum parallel transport in fermions, where the vector is the spin moving through the *B*-field space, the geometric phase is half of that [27].

Let us assume that there are two oscillations around the perpendicular axes *x* and *y* with a time dependent angular velocity in the form

31 $$\begin{aligned} (\varOmega _x, \varOmega _y) = \left( A_x \cos (\omega _x t), A_y \cos (\omega _y t + b_0)\right) . \end{aligned}$$

Integrating the expressions with respect to time, the accumulated phase as a function of time is

32 $$\begin{aligned} a_x(t)= & {} \frac{1}{\omega _x}A_x \sin (\omega _x t),\quad \text {and}\nonumber \\ a_y(t)= & {} \frac{1}{\omega _y}A_y \sin (\omega _y t + b_0), \end{aligned}$$

where $\omega _x$ and $\omega _y$ are the angular frequencies of the oscillations, $A_x$ and $A_y$ are the peak angular velocities of the spin precession around the respective axis, and $b_0$ is the difference in their phases at time $t = 0,$ which corresponds to the end of the magnetic pulse used to store the muons on a stable orbit. The peak angular velocities,

33 $$\begin{aligned} A_B&= -\frac{ea}{m}B_{\textrm{max}}, \end{aligned}$$

34 $$\begin{aligned} A_E&= \frac{e}{mc} \left( \frac{1}{\gamma ^2 - 1} - a\right) \left( \vec {\beta }\times \vec {E}\right) _{\textrm{max}}, \end{aligned}$$

of the spin precession are proportional to the amplitude of oscillation of the EM field in the reference frame of the particle.

In the case of small oscillations, the surface of the unit sphere can be approximated with a plane and the enclosed solid angle can be approximated with the area enclosed by the curves. The area under parametric curves,

35 $$\begin{aligned} {\mathcal {A}}(t) = \frac{1}{2}\int (a_x \dot{a}_y - a_y \dot{a}_x)dt, \end{aligned}$$

is calculated using Green’s theorem. In the case where $\omega _x \ne \omega _y$ one obtains

36 $$\begin{aligned} {\mathcal {A}}(t; \omega _x, \omega _y, b_0)= & {} \frac{1}{2} \frac{A_x^{\mathchoice{}{}{\scriptscriptstyle }{}}A_y^{\mathchoice{}{}{\scriptscriptstyle }{}}}{\omega _x \omega _y} \int \left( \omega _y \cos (\omega _y t + b_0)\sin (\omega _x t)\right. \nonumber \\{} & {} \left. - \omega _x \cos (\omega _x t)\sin (\omega _y t + b_0)\right) dt \nonumber \\= & {} \frac{1}{4}\frac{A_x^{\mathchoice{}{}{\scriptscriptstyle }{}}A_y^{\mathchoice{}{}{\scriptscriptstyle }{}}}{\omega _x \omega _y}\left[ \frac{\omega _x - \omega _y}{\omega _x + \omega _y}\cos ( (\omega _x + \omega _y)t + b_0)\right. \nonumber \\{} & {} \left. - \frac{\omega _x + \omega _y}{\omega _x - \omega _y} \cos ( (\omega _y - \omega _x)t + b_0)\right] , \nonumber \\ \end{aligned}$$

for the integral, which for resonant oscillations, $\omega = \omega _x = \omega _y,$ is

37 $$\begin{aligned} {\mathcal {A}}(t; \omega , b_0) = -\frac{t}{2\omega }A^{\mathchoice{}{}{\scriptscriptstyle }{}}_x A_y^{\mathchoice{}{}{\scriptscriptstyle }{}}\sin (b_0), \end{aligned}$$

resulting in an angular velocity

38 $$\begin{aligned} {\dot{\mathcal {A}}}(\omega , b_0) = -\frac{1}{2\omega }A^{\mathchoice{}{}{\scriptscriptstyle }{}}_x A_y^{\mathchoice{}{}{\scriptscriptstyle }{}}\sin (b_0). \end{aligned}$$

Another approach to obtain Eq. (37) is by using the method of averages and performing a second-order approximation of the exact Thomas-BMT equation, as done in the works of Carli and Haj Tahar [20, 28, 29].

By using Eqs. (36) and (37) one can calculate the phase accumulation as a function of time in the case of two periodic oscillations along the perpendicular axes. It can be seen that the geometric phase becomes larger with decreasing differences between the frequencies of the two oscillations. In the case of equal frequencies, the phase accumulation is linear with time and proportional to the product of the peak angular velocities of the spin precession around the two axes. In the case of equal frequencies, the geometric phase is zero when the two oscillations are in phase $(b_0 = 0)$ and is maximal when they are out of phase $(b_0 = \pi /2).$ The validity of geometrical phase calculations was verified by Monte Carlo simulations and is presented in Appendix A.4.

An example of a potential geometric-phase effect for the muon EDM experiment is resonant oscillations of the spin around the longitudinal and radial axes due to the cyclotron motion of muons in the electric field for the frozen-spin technique. This can happen when the centre of the muon’s orbit is offset from the centre of the electric field, combined with an angular misalignment of the axis of the coaxial electrodes and that of the solenoid field, as outlined in Eq. (23). The angular misalignment would lead to oscillations in $E_z$ in the rest frame of the muon and the offset of the orbit to a changing $E_\rho .$ Despite the null net $(g-2)$ precession over a cycle and null net precession due to the $E_z$ component in the muon reference frame, indicated in Eq. (27), small oscillations around the *z*- and $\rho$- axes will occur at the cyclotron angular frequency $\omega _{\textrm{c}},$ potentially leading to a systematic effect discussed in Appendix A.4.

### Other sources of spin precession

Other effects that could lead to a precession of the spin come from the muon motion in the curvature of space-time due to Earth’s gravitational field, and a possible influence of synchrotron radiation. Although these effects are negligible we have included their estimates for completeness.

#### Gravity

There are two effects of gravity that lead to a spin precession that could mimic an EDM. The direct contribution [30, 31],

39 $$\begin{aligned} \varOmega _{\textrm{GR}} = \frac{2\gamma +1}{\gamma +1}\frac{\beta }{c}g_{\textrm{e}}, \end{aligned}$$

results from general relativity, where $g_{\textrm{e}}$ is the gravitational acceleration at the surface of the Earth. The second contribution is due to an effective restoring force from either the electric or magnetic field that prevents the particles from falling. The *E*-field that is necessary to counteract the gravitational attraction of the earth is

40 $$\begin{aligned} E_{\textrm{g}} = -\frac{2\gamma ^2 - 1}{\gamma } \frac{m}{e} g_{\textrm{e}}. \end{aligned}$$

The magnitude of $E_{\textrm{g}}$ for both experimental phases is below ${30}\,{\mathrm{nV/m}}.$

In the muon EDM experiment case, both the direct and indirect effects of gravity lead to angular velocity of the spin precession on the order of ${10}\,{\mathrm{nrad/s}},$ or more than seven orders of magnitude below the statistical sensitivity. The influence of gravity on the spin precession of muons in storage rings are also calculated in [32] and estimate the systematic effect at the same order of magnitude. Therefore, we consider gravitational effects as negligible and will not discuss them further.

#### Synchrotron radiation

The muons will lose energy when circulating in the storage ring due to synchrotron radiation. This will not lead directly to spin precession, but could result in depolarisation. The power emitted by synchrotron radiation can be calculated by applying the relativistic Larmor formula,

41 $$\begin{aligned} P_\gamma = \frac{1}{6\pi \varepsilon _0}\frac{e^4}{m^2c}\gamma ^2\beta _\theta B_z. \end{aligned}$$

For the Phase I and Phase II experiments this results in an average emission of ${1.46}\,{\upmu }\textrm{eV}/ {\upmu }\textrm{s}$ and ${23.0}\,{\upmu }\textrm{eV}/ {\upmu }\textrm{s},$ respectively. Such rate of reduction in the muon kinetic energy is negligible and would not lead to any measurable effect.

Synchrotron radiation can also cause gradual polarisation of the particles (Sokolov–Ternov effect) with respect to their velocity, i.e., longitudinal in our coordinate system, according to $P \approx 1 - e^{-t/\tau _{\textrm{p}}}.$ The polarisation is perpendicular to both velocity and acceleration, thus along the magnetic field responsible for the bending. The characteristic time $\tau _{\textrm{p}}$ is [33]

42 $$\begin{aligned} \tau _{\textrm{p}} = \frac{8}{5\sqrt{3}} \frac{m^2c^2\rho _0^3}{e^2\hbar \gamma ^5}. \end{aligned}$$

For the parameters of the Phase I and II experiments the characteristic time amounts to $\tau _{\textrm{p}}\approx {10^{20}}\,{\textrm{s}},$ to be compared with the typical measurement time of ${10^{-5}}\,{\textrm{s}}.$ Therefore, spin-flip synchrotron radiation is not a concern for the experiment.

## Spatial and temporal non-uniformity of the EM field

In this section, we provide calculations for specific deviations from the ideal homogeneous EM-fields, EM-field non-uniformity, that lead to false EDM signals. Such signals can be observed by AMM-induced spin precession around the radial or azimuthal axes. The latter can occur if there is a non-zero azimuthal magnetic field component in the rest frame of the particle. This requires a net current flowing through the area enclosed by the muon orbit. All electric supply current leads are designed such that the net current flow is expected to be zero in the experimental setup. Therefore, special attention is given to the sources of radial spin precession. In this context the two most significant sources of systematic effects are: (i) a *z*-component of the electric field, and (ii) a time-varying radial *B*-field component.

Another possible source of a false EDM signal that is explored is the effect of a time-variable magnetic field that leads to a longitudinal shift of the average orbit position. Finally, we derive limits on the deviations of the fields from their nominal values and their orientations, specifying requirements for the realisation of the experimental setup.

### Field non-uniformity

Muons on the nominal orbit experience only a *B*-field along the *z*-axis, of about ${3}\,{\textrm{T}},$ and a purely radial *E*-field, such that the effect of any anomalous magnetic moment is cancelled and the relative angle between spin and momentum is constant. Any deviation of the fields from this ideal configuration or from the nominal orbit induces spin motion.

In the following analysis, we exploit that arbitrary motions can be represented as a sum of oscillations around mutually perpendicular axes to describe the effects of dynamic and geometric phases. For oscillations with a period much shorter than the measurement time of several muon lifetimes, the mean of the dynamic phase around each axis would tend to zero; the accumulation of a geometric phase remains possible.

The phase accumulation due to geometric phases can be calculated using Eq. (37). We can distinguish three types of geometric phases that can be observed: (i) due to oscillations in the spin direction caused by oscillations in the *B*-field along two perpendicular axes, (ii) due to oscillations in the spin direction caused by oscillations in the *E*-field along two perpendicular axes, (iii) due to the coupling of oscillations in the *B*- and *E*-fields. The phase accumulation due to oscillations with given frequencies can be calculated using Eqs. (36) and (37) by substituting the *B*- or *E*-field oscillation amplitude in Eq. (33) or (34), respectively. A concrete analysis in the case of the Phase I muEDM experiment is given in the discussion, Sect. 5.1.

For low-frequency oscillations $\omega \ll \tau _\mu ^{-1}$ one has to consider dynamic phase accumulation as well. In this case, a systematic effect can occur if an oscillation of a field in the muon reference frame is correlated with the injection time. As the measurement variance scales with the number of detected decay positrons, which will decrease exponentially with time, earlier times will have larger weight on the final asymmetry.

For a measurement window *L*,  the weighted average of an oscillation with unit amplitude and angular frequency $\omega ,$ weighted over the number of muons at a given time *t* after injection is

43 $$\begin{aligned} W(\omega ) = \left( \int _0^L e^{-t/\tau _\mu ^*}dt\right) ^{-1} \int _0^L \cos (\omega t+b_0)e^{-t/\tau _\mu ^*} dt, \nonumber \\ \end{aligned}$$

where the first multiplier on the left-hand side is a normalisation factor. The boosted muon lifetime is $\tau _\mu ^*= \gamma \tau _\mu$ with $\tau _\mu \simeq {2.197}\,{\upmu }\textrm{s}.$ For $L \gg \tau _\mu ^*$ Eq. (43) reduces to

44 $$\begin{aligned} W_{\textrm{L}}(\omega ; b_0) = \frac{\cos (b_0) + \gamma \tau _\mu \omega \sin (b_0)}{1+(\gamma \tau _\mu \omega )^2}. \end{aligned}$$

For all further analysis we assume $b_0 = 0$ and $W_{\textrm{L}}(\omega ) = W_{\textrm{L}}(\omega ; 0),$ as we are interested in low frequency signals that could mimic an EDM.

In the case of spin precession due to the AMM coupling with the radial component of a time-varying magnetic field $B_\rho (z(t)) = \int _0^\infty A_\rho (\omega ) \cos (\omega t + b_0) d\omega$ expressed as in Eq. (12), the requirement that the angular velocity is less than a fraction *F* of the experimental sensitivity is

45 $$\begin{aligned} \frac{ea}{m}A_\rho (\omega ) W_{\textrm{L}}(\omega ) \le F{\dot{\varPsi }}, \end{aligned}$$

where $F \in (0, 1)$ is an arbitrarily chosen factor.^2^ The limit as a function of $d_\mu$ then becomes

46 $$\begin{aligned} A_\rho (\omega ) \le F \frac{1}{W_{\textrm{L}}(\omega )}\frac{2mc}{ea\hbar }\beta _\theta B_z d_\mu . \end{aligned}$$

Calculations of the limit on the radial *B*-field in the rest frame of the muon for the muEDM experiment are given in Sect. 5.1.

In cases where the radial B-field amplitude is too large muons will not be stored. In order to derive a limit on the maximum combination of amplitude and oscillation frequency, we approximate the muon motion due to the oscillating *B*-field by a harmonic oscillator, hence, the following relation between amplitude and position holds

47 $$\begin{aligned} \frac{A_\rho (\omega )}{z_{\textrm{max}}} = \partial _z A_\rho (\omega ) = \frac{\omega ^2}{\omega _{\textrm{c}}^2}\frac{B_0}{\rho _0}, \end{aligned}$$

following a similar calculation to the one used for betatron oscillations (see Eq. (9)), and assuming a constant gradient of $B_\rho$ along *z*. Therefore, the amplitude of the oscillations of the radial *B*-field are bounded according to

48 $$\begin{aligned} A_\rho (\omega ) \le z_{\textrm{max}} \frac{\omega ^2}{\omega _{\textrm{c}}^2} \frac{B_0}{\rho _0}, \end{aligned}$$

in the muon rest frame corresponding to the maximum longitudinal displacement $z_{\textrm{max}}.$ Thus, we obtain the conditions for which the muon will not be stored in the storage ring and therefore will not contribute to the measurement signal.

### Longitudinal electric field and alternating injections

Analogously to the derivation of a limit on the phase accumulation due to dynamical phases in an oscillating radial *B*-field (Eq. (46)), we can derive a limit,

49 $$\begin{aligned} E_z(\omega ) \le F \left( \frac{e}{mc} \left( \frac{1}{\gamma ^2 - 1} - a\right) W_{\textrm{L}}(\omega ) \right) ^{-1}\frac{2c}{\hbar } B_z d_\mu \end{aligned}$$

for that induced by a longitudinal *E*-field $E_z$ using Eq. (11).

The most stringent limit on $E_z$ is reached at low frequencies approaching a constant value for $\omega \rightarrow 0.$ This limit can be relaxed considerably by taking advantage of the CP-violating nature of the EDM. Alternating periodically between clockwise (CW) and counter-clockwise (CCW) particle motion in the storage ring, with otherwise identical conditions, permits cancellation of systematic effects in the measured asymmetry arising from $E_z$-induced dynamical phase accumulation. This can be achieved by switching the polarity of the currents generating the magnetic field, thus inverting the direction of the magnetic field, and correspondingly reversing the injection direction of the muons.

Being proportional to $\vec {\beta }\times \vec {B},$ the EDM signal maintains its sign and is unchanged between the alternating injection modes. The systematic effect related to a *z*-component of the *E*-field is proportional to $\vec {\beta } \times \vec {E}.$ As the *z*-axis is defined to be aligned with the direction of the main *B*-field, $E_z$ will change sign between injections and so too the systematic effect. Thus, in the ideal setup, the systematic effect will be cancelled by summing the CW and CCW signals, while the EDM signal will double. However, the spin-phase build-up due to the longitudinal electric field might be different for the two injection modes for a variety of reasons that we will explore in more detail here.

Expanding Eqs. (13) and (28), the projection of the angular velocity due to a net non-zero longitudinal electric field (along *z*) that is less than a fraction *F* of the experimental sensitivity is

50 $$\begin{aligned}{} & {} -\frac{ea}{mc} \left( 1 - \frac{1}{a(\gamma ^2 - 1)} - \frac{1}{\beta _\theta ^2}\right) \beta _\theta |E_z |\cos (\omega _z t + \varPhi _0) \biggr |_{\mathchoice{}{}{\scriptscriptstyle }{}\textrm{CCW}}^{\mathchoice{}{}{\scriptscriptstyle }{}\textrm{CW}}\nonumber \\{} & {} \quad \le F\frac{2c}{\hbar }\beta _\theta B_z d_\mu . \end{aligned}$$

The evaluation bar denotes that we take the difference of the CW and CCW signal, where the parameters $\gamma , \beta _\theta , E_z, \omega _z$ and $\varPhi _0$ take the values corresponding to the two injection modes. Note that $\beta _\theta$ and $\gamma$ are functions of the muon momentum *p*,  and $\omega _z = \omega _z(B_z, E_\rho , p).$

As a consequence, four parameters need to be kept under strict control between CW and CCW injections to fully cancel the false signal: the particle momentum distribution for CW and CCW; the spin precession angular velocity around *z*,  which is proportional to a linear combination of $B_z$ and $\beta _\theta E_\rho ;$ the average initial phase $\varPhi _0$ of the spin in the transverse plane over the ensemble of injected particles; the average longitudinal component $E_z$ along the CW and CCW trajectories. The false EDM depends on the product of these parameters, therefore one cannot constrain a given parameter independently of the others. Specific constraints are discussed in Sect. 5.

To cancel the effects of $E_z$ by alternating the direction of circulation of the muons, it is necessary to ensure that on average the particles experience similar $E_z$ at a given time after injection. This requirement not only constrains the time stability and spatial uniformity of the applied electric field, but also the initial position and time evolution of the muon orbit which define the trajectory occupied within the field.

A possible source of time dependent changes of the muon orbit comes from the weakly-focusing field. The simplest configuration of such field comprises a single current loop, where the current flows in the opposite direction to that of the main solenoid. This arrangement generates a gradient $\partial _z B_\rho ,$ which is used to store muons in the *z*-direction. In conjunction with this, a radial gradient $\partial _\rho B_z$ arises, leading to a variation of the longitudinal *B*-field as a function of the distance from the centre of the weakly-focusing coil.

If the centre of a particle’s orbit deviates from the coil centre, the particle will encounter a stronger field and a smaller turning radius in one portion of the orbit and inversely on the opposite side. Consequently, this generates a minor phase accumulation around the centre of the focusing field with each cyclotron revolution (illustrated in Fig. 7). This is an instance of a magnetron oscillation,

51 $$\begin{aligned} \omega _{\textrm{m}} = \frac{\omega _{\textrm{b}}^2}{2\omega _{\textrm{c}}}, \end{aligned}$$

which is well described for Penning traps [34].

Interestingly, the magnetron oscillation can be thought of as being caused by the difference between the cyclotron and horizontal betatron^3^ oscillations

52 $$\begin{aligned} \omega _{\textrm{c}} - \omega _{\textrm{h}}= & {} \omega _{\textrm{c}}(1 - \sqrt{1-n}) = \omega _{\textrm{c}}\frac{n}{2} + {\mathcal {O}}(n^2) \nonumber \\= & {} \frac{\omega _{\textrm{b}}^2}{2\omega _{\textrm{c}}} + {\mathcal {O}}(n^2), \end{aligned}$$

where for $n \ll 1$ the higher order terms in the Taylor expansion can be neglected.

The magnetron oscillation does not directly generate a false EDM signal, but it could lead to different positioning of CW and CCW orbits. If there is a significant field component $E_z,$ the averages over time seen by the muon for the two injections might be unequal, thus failing to cancel the systematic effect. This is discussed in greater detail in Sect. 5.2.

### Longitudinal shift of the average orbit

The systematic effects discussed so far concern the precession of the spin within the muon’s reference frame. However, radial *B*-fields, external to the weakly-focusing field, could lead to a rotation in the momentum vector, thereby generating an EDM-like signal. The magnitude of this effect can be significantly higher than the spin precession by approximately $(\gamma a)^{-1}$ times $(\approx$ 800 for the Phase I experiment).

In the storage ring, the particle orbit is in a weakly-focusing field characterised by a $\partial _z B_\rho$ gradient. While a constant external radial *B*-field would merely alter the *z*-equilibrium position of the orbit and not cause a systematic effect, a radial field with amplitude $B'_{\textrm{tr}}$ fluctuating in time could result in a drift of position, generating a systematic effect. The maximum amplitude of this longitudinal drift $d_{\textrm{l}} = B'_{\text {tr}} / \partial _z B_\rho ,$ where the prime denotes that the $B'_{\textrm{tr}}$ is the magnetic flux density in the laboratory reference frame.

The motion of the muon,

53 $$\begin{aligned} \ddot{z} = -\frac{e}{\gamma m} c \beta _\theta \left[ z \partial _z B_\rho + B'_{\textrm{tr}} \cos (\omega t) \right] , \end{aligned}$$

depends on the combined effect of the weakly focusing field $\partial _z B_\rho$ and the transient field $B'_{\textrm{tr}} \cos (\omega t)$ with the oscillation frequency $\omega =2\pi f.$ The solution of (53),

54 $$\begin{aligned} z(t) = \frac{u B'_{\textrm{tr}}}{u \partial _z B_\rho + \omega ^2} \cos (\omega t) + z_0 \cos (\omega _{\textrm{b}}t + \phi _0), \end{aligned}$$

is similar to Eq. (9), where $u = \frac{e}{\gamma m} c \beta _\theta .$ Considering only the first term with oscillations due to the transient field, and substituting $\omega _{\textrm{b}} = u\partial _z B_\rho ,$ it is convenient to define the longitudinal pitch angle,

55 $$\begin{aligned} P_{\textrm{l}} = \frac{\dot{z}_{\textrm{tr}} (t)}{c\beta _\theta } = - \frac{e}{\gamma m}\frac{\omega }{\omega _{\textrm{b}}^2 + \omega ^2}B'_{\textrm{tr}}\sin (\omega t), \end{aligned}$$

as the ratio of longitudinal to azimuthal velocity. The time derivative,

56 $$\begin{aligned} \dot{P}_{\textrm{l}} = - \frac{e}{\gamma m}\frac{\omega ^2 }{\omega _{\textrm{b}}^2 + \omega ^2}B'_{\textrm{tr}}\cos (\omega t), \end{aligned}$$

yields the rate of change relevant for calculating the potential systematic effect. We calculate an upper limit for this effect due to longitudinal drift of the muon orbit, by taking the weighted average of $\dot{P}_{\textrm{l}},$

57 $$\begin{aligned} \dot{P}_{\textrm{W}} = \frac{e}{\gamma m}\frac{\omega ^2 }{\omega _{\textrm{b}}^2 + \omega ^2}B'_{\textrm{tr}}W_{\textrm{L}}(\omega ) \le F\frac{2c}{\hbar }\beta _\theta B_z d_\mu . \end{aligned}$$

This results in a limit,

58 $$\begin{aligned} B'_{\textrm{tr}} \le F \left[ \frac{e}{\gamma m}\frac{\omega ^2}{\omega _{\textrm{b}}^2 + \omega ^2} W_{\textrm{L}}(\omega ) \right] ^{-1} \frac{2c}{\hbar }\beta _\theta B_z d_\mu , \end{aligned}$$

as a function of $\omega$ and the betatron frequency $\omega _{\textrm{b}}.$ Specific limits for this orbit-drift-effect, which could result from the magnetic kick and thereby induced eddy currents are derived and discussed in Sect. 5.

Note that Eq. (58) gives the limit on $B'_{\textrm{tr}}(\omega )$ for a specific $\omega .$ The residual tail from the magnetic kick will contain a wide frequency spectrum. Therefore, the spin rotation due to the integral over $\omega ,$

59 $$\begin{aligned} \frac{e}{\gamma m}\int _0^\infty \frac{\omega ^2 }{\omega _{\textrm{b}}^2 + \omega ^2}B'_{\textrm{tr}}(\omega )W_{\textrm{L}}(\omega )\, d\omega \le F\frac{2c}{\hbar }\beta _\theta B_z d_\mu , \end{aligned}$$

has to be constrained.

We can also derive limits on $B'_{\textrm{tr}}(\omega )$ such that

$$\begin{aligned} \max (z_{\textrm{tr}}(t)) \le z_{\textrm{max}}, \end{aligned}$$

where $z_{\textrm{max}}$ is defined in Sect. 4.1 as the maximum longitudinal displacement for stored muons, similar to Eq. (48) that gives the relationship between *B*-field oscillation amplitude and frequency in the muon rest frame for stored muons. With Eq. (54) for $\cos (\omega t) = 1$ and $\cos (\omega _{\textrm{b}} t + \phi _0) = 1,$ we obtain

60 $$\begin{aligned} B'_{\textrm{tr}} \le (z_{\textrm{max}} - z_0) \frac{\gamma m }{ec\beta _\theta } (\omega _{\textrm{b}}^2 + \omega ^2). \end{aligned}$$

For low frequencies $\omega \rightarrow 0$ the equation reduces to $z_{\textrm{max}} - z_0 \ge B'_{\textrm{tr}} / \partial _zB_\rho ,$ leading to the expected result that the control of the maximum displacement due to external radial fields can be established through the strength of the weakly focusing field.

## Discussion

Following from the analysis of Sect. 4, we have carried out Monte Carlo studies to identify and investigate several severe systematic effects that, if not properly controlled, would limit the maximum achievable sensitivity for the muon EDM experiment. These are effects related to the accumulation of geometrical phases. Effects arising from a non-zero average electric field along the solenoid axis in the muon reference frame were introduced in Sect. 4.1, and results from the associated studies are presented in Sects. 5.1 and 5.2. The transverse precession of the orbit around the axis of symmetry of the weakly-focusing field (magnetron oscillations) were shown in Sect. 4.1 and the case study for the Phase I experiment is given in Sect. 5.2.2. Finally, the limits due to the movement of the average orbit due to a changing external radial magnetic field, theoretically explored in Sect. 4.3, are shown in Sect. 5.3.

All calculations were performed assuming the parameters of the Phase I muon EDM experiment, unless explicitly stated otherwise. Phase I aims to achieve a sensitivity of $\sigma (d_\mu )= {3\times 10^{-21}}\,{e\!\cdot \!\textrm{cm}}.$ We choose to set limits for each individual systematic effects to a fraction of the experimental sensitivity, $F = 1/4.$ The muon momentum is $p = {28}\,{\textrm{MeV}/\textit{c}}$ corresponding to $\beta _\theta = 0.26.$ The main magnetic field is $B_z = {3}\,{\textrm{T}},$ and the weakly focusing field has a gradient $\partial _zB_r = {80}\,{\mathrm{mT/mm}}$ at the radius of the nominal orbit. Throughout this discussion we assume a worst-case scenario where all time-dependent systematic effects are correlated to the injection time and the initial parameters (polarisation, momentum, etc.) have a systematic offset between CW and CCW injections.

### Limits on geometrical phases and EM field non-uniformity

**Fig. 3:**
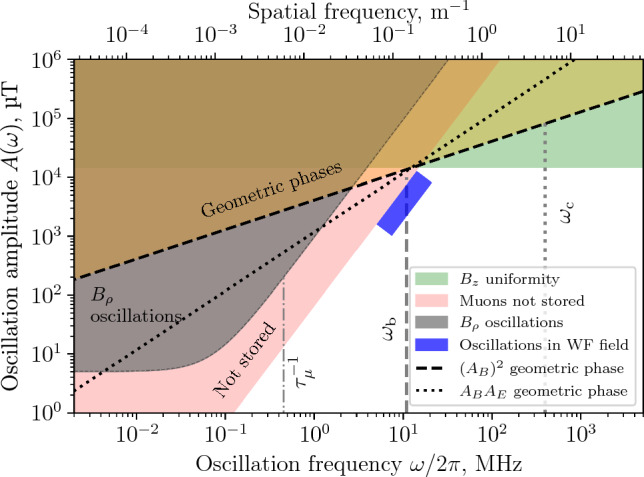
Limits deduced for a worst case false EDM signal in the Phase I experiment due to resonant *B*-field oscillations as a function of the oscillation frequency and its amplitude (maximum deviation from the nominal) in the muon reference frame. The dash-dot line corresponds to the inverse of the muon decay time $({2.2}\,{\upmu }\textrm{s}),$ the dashed vertical line shows the expected angular velocity corresponding to the betatron oscillations $({150}\,{\textrm{ns}}$ period) and the dotted one to the cyclotron oscillations $({2.5}\,{\textrm{ns}}$ period). The blue rectangle shows the possible values for the weakly-focusing field oscillation amplitude and frequency range. The second abscissa shows the spatial frequency, giving the number of periods per meter travelled by the muons and calculated as $f = \omega / (2\pi \beta c)$

We first deal with geometrical phases induced by non-uniformity in the *B*-field according to the framework set up in Sect. 4.1. To place limits on the field non-uniformity inducing geometrical phases we assumed the worst case where the oscillations are maximally out of phase and the oscillations around the two perpendicular axes have the same amplitude. The limits on the amplitude of the oscillation as a function of the oscillation frequency are shown in Fig. 3 (depicted as the coloured area above the $(A_B)^2$ dashed line). The calculations were performed using Eq. (38) requiring that the rate of geometrical phase accumulation is a fraction *F* of the experimental sensitivity

61 $$\begin{aligned} {\dot{\mathcal {A}}}(\omega ) = \frac{A_x A_y}{2\omega } \sin (b_0) \le F\frac{2c}{\hbar }\beta _\theta B_z d_\mu , \end{aligned}$$

where $A_x = A_y = -eaB_{\textrm{max}}/m$ and the initial phase $b_0 = \pi /2.$ For reference, the region of expected betatron oscillation amplitudes and frequencies is presented with a blue rectangle.

The geometric phase accumulation due to a combined *B*-field and *E*-field non-uniformity is shown in the same figure with a dotted line labelled $A_B A_E$ at the level of 0.5% of the radial *E*-field required for the frozen spin state, corresponding to $E_{\textrm{max}} = {1.4}\,{\mathrm{kV/m}}.$ The limit was calculated using Eq. (61), where $A_x = -eaB_{\textrm{max}}/m$ and $A_y$ is given by Eq. (34).

The main source of a time-variable radial magnetic field is the split coil pair used to kick the particles into a stable orbit. The nominal current pulse is a half-sine pulse with ${100}\,{\textrm{ns}}$ half-period, producing a radial magnetic field of a few hundred ${\upmu }\textrm{T}$ peak in the storage region. A real pulse will not follow exactly the half-sine and will exhibit ringing with a finite decay time. Another source of a slowly decaying radial magnetic-field components could be eddy currents induced by this pulse in the electrode system or the bore of the main solenoid.

For low-frequency oscillations, especially when tending to zero, one has to consider dynamic phase accumulation, where the limits on the amplitude of the *B*-field oscillations are given by Eq. (46). The limits from this source of systematic effects are given in Fig. 3 with a grey area labelled “$B_\rho$ oscillations”. The same reasoning can be applied to the azimuth component of the magnetic field, but the expected amplitude of the $B_\theta$ oscillations is negligible.

Not all combinations of oscillation frequency and amplitude of the *B*-field lead to stored muons. Using Eq. (48) and imposing that the oscillation amplitude of the muon $z_{\textrm{max}} \le {50}\,{\textrm{mm}}$ we obtain the red exclusion area (labelled “Not stored”) in Fig. 3. The ${50}\,{\textrm{mm}}$ limit on the longitudinal oscillation amplitude is due to the positioning of the split coil pair of the magnetic kicker. Particles would only be able to be stopped within the region where the radial magnetic field generated by the current pulse is such that the Lorentz force $e c \vec {\beta } \times \vec {B}$ counteracts the longitudinal motion, which is in between the two current loops.

Systematic effects can only be caused by $B_\rho$-field components, when limiting our considerations to the *B*-field, as only these will lead to an EDM-like spin precession. However, dynamic phase effects related to the electric field can be significant if the muon orbit deviates from circular and the electrode system is tilted with respect to the central solenoid axis. In this case, the mean longitudinal component of the electric field over a cyclotron rotation would not be zero, as concluded from Eq. (27). Assuming a ${1}\,{\textrm{mrad}}$ tilt of the electrode system, the eccentricity, $e=\sqrt{1-a^2/b^2},$ where *a* and *b* are the semi-major and semi-minor axis of the ellipse, of the muon orbit has to be kept below $e\le 0.1.$ This corresponds to a uniformity of the *z*-component of the *B*-field within $\pm {15}\,{\textrm{mT}}$ (depicted as “$B_z$ uniformity” in Fig. 3). Effects of $B_z$ non-uniformity resulting in, e.g., magnetron oscillation are discussed in Sect. 4.2.

From the results shown in Fig. 3, we can see that the geometric phases that are caused by *B*-field variation in time impose weaker limits compared to other uniformity considerations. The observation of a false EDM-signal due to oscillations of the spin around the radial axis is also not possible, as the oscillations with significant amplitude would not correspond to stored muons. The unavoidable betatron oscillations due to the weakly-focusing field do not violate the calculated constraints and will not lead to a significant false EDM-signal.

Nevertheless, the presence of a radial *B*-field that is external to the weakly-focusing field would cause a shift in the longitudinal position of the average muon orbit. The systematic effects related to a possible shift of the orbit equilibrium position with time are expanded upon in Sect. 5.3.

**Fig. 4:**
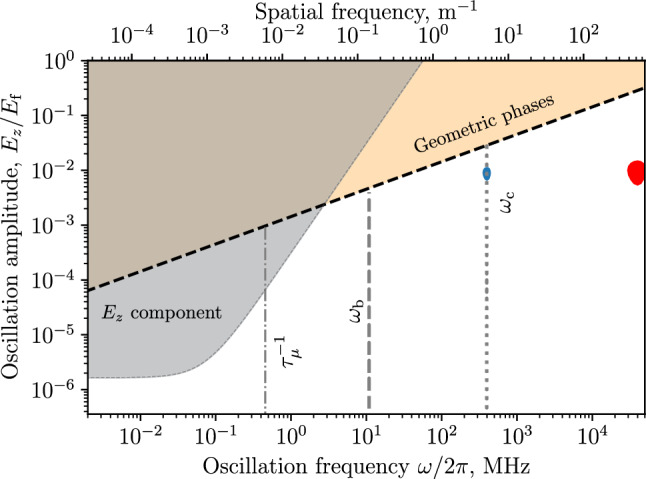
The worst case limits on the *E*-field oscillation frequency and amplitude (maximum deviation from the nominal) given as a fraction of $E_{\textrm{f}}.$ The dashed vertical line shows the angular velocity corresponding to the betatron oscillations and the dotted one to the cyclotron oscillations. The dashed-dotted vertical line corresponds to the inverse muon lifetime and, coincidentally, roughly to the $g-2$ precession frequency without frozen-spin. The grey area is the limit of the longitudinal *E*-field as a result of the dynamical phase accumulation. The blue and red shapes show the parameter space for tilted electrodes and displaced muon orbit for solid or striped electrodes, respectively

Figure 4 shows the limit derived from the geometric phase due to oscillations of the radial and longitudinal electric-field components in the muon reference frame, using Eq. (61), where $A_x = A_y = A_E$ is defined by (34).

The expected false EDM signal due to $E_z$ oscillations was calculated using Eq. (49). The limit on $E_z$ uniformity is shown in Fig. 4 as grey area. All limits were calculated considering only a single direction of circulation of the muons, i.e., only CW or CCW.

The analysis of the electric field uniformity shows that the maximum allowed non-uniformity at the betatron frequency is $0.4\%$ of the electric field $E_{\textrm{f}}$ required to freeze the spin to the momentum. Such a time-dependent variation of the electric field in the muon reference frame can occur due to the fringe field from the end regions of the electrodes. Studies using finite-element methods (FEM) show that this effect can be mitigated by using sufficiently long, i.e. ${500}\,{\textrm{mm}},$ electrodes, which would result in negligible fringe fields $(E_z \le {0.02}\,{\mathrm{V/m}})$ in the storage region.

The resonance between radial and longitudinal *E*-field oscillations caused by a tilt in the electrode system and a displacement of the muon orbit could cause the build up of geometric phases (discussed also in Sect. 3.5). Assuming a ${1}\,{\textrm{mrad}}$ tilt of the electrodes with respect to the central axis of the solenoid the muons will experience an oscillating field $E_z$ at the cyclotron frequency with amplitude ${0.3}\,{\textrm{kV}}$ due to the projection of the radial electric field along the *z*-axis. If the orbit center is displaced from the central axis of the electrodes, the muons will also experience an oscillating radial *E*-field at the cyclotron frequency. The amplitude of the oscillation depends on the magnitude of the displacement and is approximately ${10}\,{\textrm{kV}}$ for ${1}\,{\textrm{mm}}$ displacement. One can show that the combined effect of the tilt and displacement results in a geometric phase build-up equivalent to both oscillations having an amplitude of ${1.7}\,{\textrm{kV}}.$ This equivalent case $({1.7}\,{\textrm{kV}}$ amplitude at $\omega _{\textrm{c}})$ is contained in the blue region in Fig. 4 (lower limit of $E_z / E_{\textrm{f}}).$ Orbit displacement of ${3}\,{\textrm{mm}}$ would result in ${30}\,{\textrm{kV}}$ radial *E*-field oscillation, which, combined with the ${0.3}\,{\textrm{kV}}$ $E_z$ oscillation, is equivalent to both having ${3}\,{\textrm{kV}}$ amplitude. This case is also contained in the blue region (upper limit of $E_z / E_{\textrm{f}}).$ Note that both the tilt and the displacement used for the calculation are larger than the limits imposed by consideration of other sources of systematic effects. This means that even in the worst-case scenario the accumulation of geometric phase due to a displacement of the muon orbit with respect to the centre of the electric field is negligible.

Another scenario for the generation of geometric phases is explored by assuming the same tilt, but an electrode system composed of individual discrete wires instead of a solid cylinder, discussed in Appendix B. This would create non-uniformity in the radial electric field, which, in the muon reference frame, will oscillate with a multiple of the cyclotron frequency. The red shape in Fig. 4 shows the additional parameter space occupied in this scenario, due to the additional source of geometric phase accumulation at the given multiple of $\omega _{\textrm{c}}.$ As the limit on field amplitude of the geometric phase effect increases with increasing frequency, a setup with a segmented electrode is not excluded due to systematic considerations. Potential benefits of this electrode type are also explored in Appendix B.

**Fig. 5:**
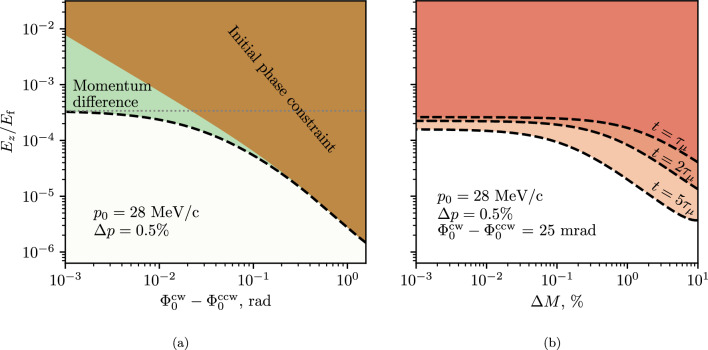
Limit on the longitudinal *E*-field, $E_z,$ when considering alternating CW/CCW injections, where the difference in mean momentum averaged over all injected muons for injections of CW and CCW beams is fixed at $\varDelta p = 0.5\%.$ **a** Shows the limit as a function of the initial phase difference $\varPhi _0^{\textrm{cw}} - \varPhi _0^{\textrm{ccw}}.$ The horizontal dashed line indicates the limit coming from $\varDelta p,$ keeping all other parameters equal between the two injection modes. The brown area is the constraint coming from the difference in initial phase at $\varDelta p = 0\%.$ The green area and its thick dashed line edge show the combined limit of the two effects. **b** Shows the limit as a function of the difference in *E*- or *B*-field for CW and CCW orbits, calculated at various times. $\varDelta M = 2 (M^{\textrm{cw}} - M^{\textrm{ccw}})/(M^{\textrm{cw}} + M^{\textrm{ccw}}),$ where $M \in \{B_z, {\tilde{E}}_\rho \},$ while the difference in initial phases was fixed at ${25}\,{\textrm{mrad}}$

### Systematic limits on a longitudinal electric field

By far the main source of a systematic effect in the frozen-spin technique would come from a non-zero *z*-component of the electric field. While this can be significantly mitigated by employing counter-rotating beams, it follows from Eq. (50) that the combination of four parameters need to be kept constant between CW and CCW injections: (i) the particle velocity $\beta _\theta ,$ (ii) the spin precession angular velocity around the longitudinal axis $\omega _z,$ which is proportional to the linear combination of $B_z$ and $\beta _\theta E_\rho ,$ (iii) the average initial phase $\varPhi _0$ of the spin in the transverse plane over the ensemble of injected particles, and (iv) the average longitudinal component $E_z$ along the CW and CCW trajectories. Note that the overall systematic effect is proportional to the product of these parameters. Therefore, improvements in the control of a single parameter are effective up to a point, after which one must constrain the rest as well. For example, even controlling $\beta _\theta ^{\textrm{cw}}$ and $\beta _\theta ^{\textrm{ccw}}$ to an excellent precision, the effect of a non-zero $E_z$ could still be significant due to differences between $\varPhi _0^{\textrm{cw}}$ and $\varPhi _0^{\textrm{ccw}}.$

To untangle the problem, first we assume that $\langle E_z^{\textrm{cw}} \rangle = -\langle E_z^{\textrm{ccw}}\rangle = E_z.$ Then we express $E_z$ from (50) as:

62 $$\begin{aligned} E_z = \frac{F {\dot{\varPsi }}}{-\frac{ea}{mc}\left[ \left( 1 - \frac{1}{a(\gamma ^2 - 1)} - \frac{1}{\beta ^2_\theta } \right) \beta _\theta \cos (\omega _z t + \varPhi _0)\right] \Big |_{\textrm{ccw}}^{\textrm{cw}}}, \nonumber \\ \end{aligned}$$

giving the maximum permissible $E_z$ that would lead to a false EDM signal equal to the threshold of $F = 1/4$ of the experimental sensitivity.

The first parameter $\beta _\theta$ places constraints on the level of control of the difference in momentum $\varDelta p = p^{\mathchoice{}{}{\scriptscriptstyle }{}\textrm{CW}}- p^{\mathchoice{}{}{\scriptscriptstyle }{}\textrm{CCW}}$ for the two injection schemes. For the Phase I muon EDM experiment, using ${28}\,{\textrm{MeV}/\textit{c}}$ surface muons, it is reasonable to aim for momentum control that ensures no more than $\varDelta p = 0.5$% difference in the mean value of the momentum for injections of CW and CCW beams. Note that such a difference not only leads to a difference in $\beta _\theta ,$ but also in $\omega _z.$ Thus, we need to specify the limit on $E_z$ at a given time *t*. Here we conservatively choose a time around the end of the measurement $t = 5\tau _\mu$ or ${11}\,{\upmu }\textrm{s}.$ With this constraint, it is possible to place a limit on the difference between the initial phases $\varPhi _0^{\textrm{cw}}$ and $\varPhi _0^{\textrm{ccw}}.$ The maximum permitted longitudinal *E*-field as a function of the difference of initial phases is shown in Fig. 5a. A further reduction of the initial phase difference below 25 mrad will become ineffective as it reaches the limit set by the control of the momentum.

A determination of the initial phase can be achieved by a dedicated $g-2$ precession measurement, by observing the spin precession without an electric field. We may tune the value of the initial phase using a Wien filter in the secondary beamline. The stability of the radial electric field in the muon reference frame can be measured by applying $E=-E_{\textrm{f}}$ to the electrodes and measuring the frequency stability of $\varOmega ^{\mathchoice{}{}{\scriptscriptstyle }{}\text {AMM}}\simeq 2ea|\vec {B} |/ m.$

The limits on $\varPhi _0^{{\textrm{cw}}} - \varPhi _0^{{\textrm{ccw}}}$ and $\varDelta p$ constrain the initial condition for muon storage. However, in the case where the spin is not perfectly frozen, the spin phase will evolve with time. For the phase accumulation to remain the same for both injections, the radial electric field in the muon reference frame must be the same within some limits. The same holds for the longitudinal *B*-field as $\omega _z$ depends on the linear combination between $B_z$ and ${\tilde{E}}_\rho .$

The maximum longitudinal *E*-field component permissible in the muon reference frame as a function of the difference in $B_z$ or ${\tilde{E}}_\rho$ is shown in Fig. 5b, where $\varDelta M = 2 (M^{\textrm{cw}} - M^{\textrm{ccw}})/(M^{\textrm{cw}} + M^{\textrm{ccw}}),$ and $M \in \{B_z, {\tilde{E}}_\rho \}.$ The calculation was performed at the fixed limit of the momentum and the initial phase difference for the two injection schemes, $0.5\%$ and 25 mrad, respectively. The imperfect cancellation of the systematic effect due to a longitudinal electric field is proportional to $\cos (\omega _z t),$ hence a function of storage time. Assuming that the longitudinal electric field is strictly proportional to the radial *E*-field and hence the longitudinal *B*-field, an improved control of better than $\varDelta M \le 0.01\%$ will not further reduce the effect, as the limiting factor becomes the initial phase and the momentum difference.

Finally, under the assumption that the longitudinal *E*-field is unchanged between injection modes, we can place a limit of $E_z \le 10^{-4}E_{\textrm{f}}.$ This corresponds to $\varDelta p = 0.5\%,$ $\varPhi _0^{{\textrm{cw}}} - \varPhi _0^{{\textrm{ccw}}} = {25}\,{\textrm{mrad}}$ and $\varDelta {\tilde{E}}_\rho = 0.1\%.$ The stability of the *B*-field can be controlled to an order of magnitude better than $\varDelta B_z = 0.01\%$ $({300}\,{\upmu }\textrm{T})$ and does not contribute significantly to this limit. One could tighten the limit on some parameter, in an attempt to relax the limit on $E_z,$ however, the contribution of the others will then become more significant. For example, reducing $\varDelta p$ by an order of magnitude to 0.05% and $\varPhi _0^{{\textrm{cw}}} - \varPhi _0^{{\textrm{ccw}}}$ by 2.5 times to ${10}\,{\textrm{mrad}}$ leads to a relaxed limit of $E_z \le 5\times 10^{-4}E_{\textrm{f}}.$

**Fig. 6:**
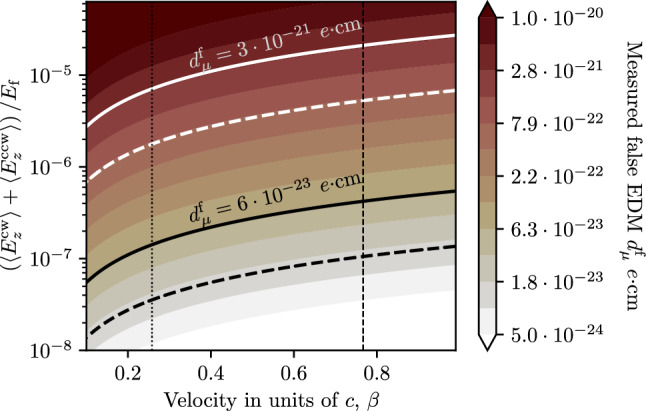
The measured false EDM $d_\mu ^{\textrm{f}}$ due to difference in the average electric field in the longitudinal direction between CW and CCW injections. The solid horizontal line shows the target sensitivity and the dashed horizontal line is one quarter of that value. The vertical dotted line is at $\beta = 0.256$ (Phase I) and the vertical dashed line is at $\beta = 0.770$ (Phase II)

#### Variation in the longitudinal electric field

So far, we have assumed that the absolute value of the average $E_z$ over time and over all measured muons is the same for the two injection modes, or $\langle E^{\textrm{cw}}_z \rangle = -\langle E^{\textrm{ccw}}_z \rangle ,$ which is only true if the muon trajectories for CW and CCW overlap perfectly. The false EDM signal measured as a function of the muon velocity and the difference in the average longitudinal electric field in the muon reference frame is shown in Fig. 6. For Phase I of the experiment this difference should be limited to $\langle E^{\textrm{cw}}_z \rangle + \langle E^{\textrm{ccw}}_z \rangle \le 2\times 10^{-6}E_{\textrm{f}}.$

**Fig. 7:**
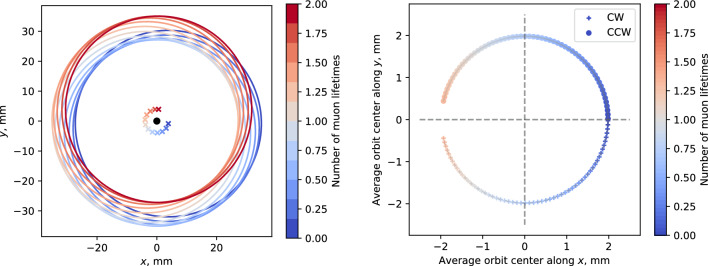
Precession of the muon orbit around the centre of the weakly-focusing field (magnetron oscillations). Left: Time evolution of the orbit of a single muon (line) and its centre (cross) around the centre of the focusing field (dot). Right: Precession of the mean centre of the orbits of an ensemble of muons for CW and CCW injections

Taking into account all considerations, we can place a limit on the maximum *z*-component of the electric field allowed in the muon reference frame of $E_z\le 10^{-4}E_{\textrm{f}},$ or approximately $\langle E_z^{\textrm{cw, ccw}} \rangle \le ~{28}\,{\mathrm{V/m}}$ and its maximum change between the two injections $\left| \langle E^{\textrm{cw}}_z \rangle + \langle E^{\textrm{ccw}}_z \rangle \right| \le {0.56}\,{\mathrm{V/m}}$ for the Phase I muon EDM experiment. The corresponding values for the Phase II experiment are $\langle E_z^{\textrm{cw, ccw}} \rangle \le ~{2.9}\,{\mathrm{V/m}}$ and $\left| \langle E^{\textrm{cw}}_z \rangle + \langle E^{\textrm{ccw}}_z \rangle \right| \le {0.15}\,{\mathrm{V/m}}.$

#### Slow drift of the muon orbit

One possible reason for differences between the absolute values of $\langle E_z^{\textrm{cw}}\rangle$ and $\langle E_z^{\textrm{ccw}} \rangle$ is the magnetron oscillation of the muon, discussed in Sect. 4.2. For a typical Phase I cyclotron frequency of $\omega _{\textrm{c}} = {2.47}\,{\mathrm{rad/ns}}$ and betatron frequency $\omega _{\textrm{b}} = {0.073}\,{\mathrm{rad/ns}},$ the magnetron frequency is $\omega _{\textrm{m}} = {0.11\times 10^{-3}}\,{\mathrm{rad/ns}}$ corresponding to a period of ${5.8}\,{\upmu }\textrm{s}.$

We explore this effect through a Monte Carlo simulation using Geant4. The initial *z*-position of each particle was drawn from a Gaussian distribution with mean ${0}\,{\textrm{mm}}$ and ${10}\,{\textrm{mm}}$ standard deviation. The transverse position was uniformly distributed along a circle with radius equal to the nominal radius of the muon storage orbit, i.e. ${31}\,{\textrm{mm}},$ and centred at $(x_0, y_0) = (2,0)\pm (3,3)$ mm. The momentum of the particles is sampled from Gaussian distribution with mean and standard deviation ${(28.0\pm 0.3)}\,{\textrm{MeV}/\textit{c}}.$ The sampling approximates conditions in which the muons are injected in the experiment slightly off the nominal orbit. The simulations were performed with positive and negative *B*-field to study the orbit precession for the CW and CCW cyclotron motion. The mean position of the centre of the orbits as a function of time is shown in Fig. 7.

The observed magnetron oscillation period is consistent with the prediction of ${5.8}\,{\upmu }\textrm{s}.$ From Eq. (51), the sign of the magnetron oscillation follows the sign of $\omega _{\textrm{c}},$ which can be seen in Fig. 7 as well. The direction of the drift of the orbit center changes between CW and CCW circulation. Another observation from the simulations performed is that, even though the initial muons start with random displacements from the central axis of the weakly focusing field and a distribution of momenta, the mean orbit centre follows a circular path with ${2}\,{\textrm{mm}}$ radius starting at the (2, 0) mm position. Thus showing, that the mean position of the muon orbits at the beginning of the measurement (after the end of the nominal magnetic kick) is sufficient to describe the mean magnetron motion.

The magnetron oscillation does not directly lead to a systematic effect, however, it might invalidate the assumption that $\langle E_z^{\textrm{cw}} \rangle = - \langle E_z^{\textrm{ccw}}\rangle$ as the muons in the two injection schemes could sample a different volume and therefore a different longitudinal *E*-field. This effect can be mitigated by ensuring that the mean centre of the orbits coincides with the central axis of the weakly focusing field. Another mitigation strategy is to limit the $\partial _x E_z$ and $\partial _y E_z$ gradients, such that the longitudinal electric field is sufficiently uniform. One can estimate the limits on those gradients by approximating

63 $$\begin{aligned} \langle E^{\textrm{cw}}_z \rangle= & {} E^{\textrm{cw}}_0 + \int _{0}^{2\pi }\left[ (x_0 + \rho _0\cos \phi ) \partial _x E_z \right. \nonumber \\{} & {} \left. + (y_0 + \rho _0 \sin \phi )\partial _y E_z \right] d\phi \nonumber \\= & {} E^{\textrm{cw}}_0 + x_0\partial _x E_z + y_0\partial _y E_z , \end{aligned}$$

where $(x_0, y_0)$ is the offset of the orbit center from the central electrode axis and $E^{\textrm{cw}}_0$ is a constant term in $E_z(\vec {r}).$ Then

64 $$\begin{aligned} \langle E_z^{\textrm{cw}} \rangle + \langle E_z^{\textrm{ccw}}\rangle = \delta _x\,\partial _x E_z + \delta _y\,\partial _y E_z , \end{aligned}$$

where $\delta _x = |x^{\textrm{cw}}_0|- |x^{\textrm{ccw}}_0|$ and $\delta _y = |y^{\textrm{cw}}_0 |- |y^{\textrm{ccw}}_0|,$ and noting that $E_0^{\textrm{cw}} = -E_0^{\textrm{ccw}}$ due to the coordinate axis *z* following the *B*-field direction.

Assuming a maximum systematic offset between the mean centre of CW and CCW orbits of $\delta _x = \delta _y = {1}\,{\textrm{mm}}$ implies that $(\partial _x E_z, \partial _y E_z) \le {0.56}\,{\mathrm{kV/m/m}}$ for Phase I. Given in terms of a fraction of the frozen-spin field this is $(\partial _x E_z, \partial _y E_z) \le 0.2\% E_{\textrm{f}}{\textrm{m}^{-1}}.$

### Systematic limits on a transient radial magnetic field

As elaborated on in Sect. 4.3, a transient radial magnetic field with angular frequency $\omega$ and amplitude $B'_{\textrm{tr}}$ could introduce a systematic effect by rotating the momentum vector at a rate $\dot{P}_{\textrm{l}}$ around the radial axis, thus mimicking an EDM signal. The constraints on a single frequency oscillating radial magnetic field with oscillation amplitude $B'_{\textrm{tr}}$ as a function of its angular frequency $\omega$ are calculated using Eq. (58), where we require that $\dot{P}_{\textrm{W}} \le F {\dot{\varPsi }} = {\dot{\varPsi }}/4,$ and where $\dot{P}_{\textrm{W}}$ is the $\dot{P}_{\textrm{l}}$ weighted over the exponential decay of the muons.

**Fig. 8:**
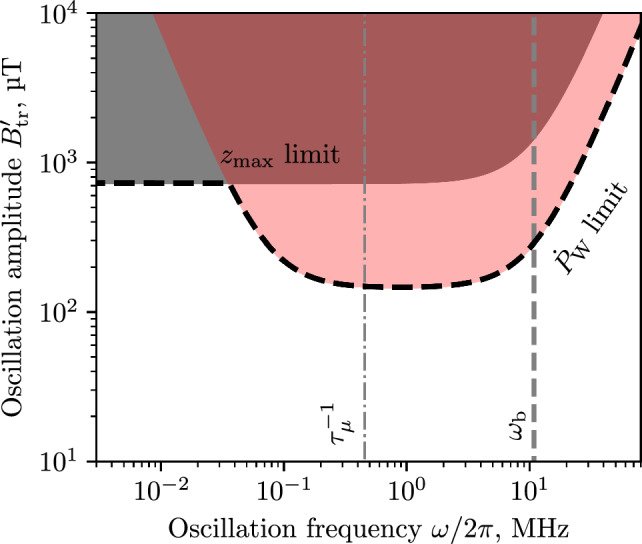
Limit on the amplitude of a transient radial magnetic field $B'_{\textrm{tr}}$ with oscillation frequency $\omega /2\pi .$ For frequencies in the range between ${200}\,{\textrm{kHz}}$ and ${10}\,{\textrm{MHz}}$ the limit reaches a plateau at ${140}\,{\upmu }\textrm{T}.$ The betatron frequency $\omega _{\textrm{b}}$ is shown with a vertical dashed line, and the rest-frame muon lifetime $\tau _\mu$ is shown with a dash-dot line. The grey exclusion area is calculated using Eq. (60) for $z_{\textrm{max}} = {50}\,{\textrm{mm}}$ and $z_0 = {40}\,{\textrm{mm}}.$ The thick black dashed line shows the combined limits from $z_{\textrm{max}}$ and $\dot{P}_{\textrm{W}}$

Using Eq. (60) we deduce a limit by requiring that the transient magnetic field does not lead to a too large displacement of the muon along *z* leading to a loss of the muons. The maximum amplitude of longitudinal oscillations, $z_{\textrm{max}} = {50}\,{\textrm{mm}},$ is defined by the maxima of the radial magnetic field in the weakly focusing field area. Limiting the maximum acceptable amplitude due to the betatron oscillations to $z_0 = {40}\,{\textrm{mm}}$ leaves ${10}\,{\textrm{mm}}$ for the peak displacement due to disturbances by $B'_{\textrm{tr}}.$ The limits on the oscillation amplitude of $B'_{\textrm{tr}}$ as a function of its frequency are shown in Fig. 8. For frequencies in the range between ${200}\,\textrm{kHz}$ and ${10}\,{\textrm{MHz}}$ the limit reaches a plateau at ${140}\,{\upmu }\textrm{T}.$ This corresponds to longitudinal oscillations of the mean orbit with ${0.2}\,{\textrm{mm}}$ amplitude.

The limit presented in Fig. 8 is valid in the case of an external *B*-field oscillating at a single frequency. In reality, i.e. the residual transient field from the magnetic kick, the signal will contain a spectrum of frequencies. One has to then consider the integral over the effect of $B'_{\textrm{tr}}(\omega )$ as in Eq. (59). For simplicity we take the ratio

65 $$\begin{aligned} \frac{B'_{\textrm{tr}}(\omega )}{B'_{\textrm{L}}(\omega )} = \frac{\dot{P}_{\textrm{W}}(\omega )}{F{\dot{\varPsi }}}, \end{aligned}$$

where $B'_{\textrm{L}}(\omega )$ is the external *B*-field that satisfies the equation $\dot{P}_{\textrm{W}}(\omega ) = F{\dot{\varPsi }},$ and ${\dot{\varPsi }}$ is the limit angular velocity of spin precession by an EDM equal to the statistical sensitivity of the experiment. This ratio can be thought of as what fraction of the limit angular velocity is induced by an external field $B'_{\textrm{tr}}$ at a given oscillation frequency $\omega .$ The integral of the ratio must be less than 1 to limit the combined effect of a signal with a spectrum of frequencies,

66 $$\begin{aligned} \int _0^\infty \frac{B'_{\textrm{tr}}(\omega )}{B'_{\textrm{L}}(\omega )}\,d\omega \le 1. \end{aligned}$$

The relationship $B'_{\textrm{tr}}(\omega )$ can be obtained from the inverse Fourier transform of $B^{\mathchoice{}{}{\scriptscriptstyle }{}\textrm{K}}_\rho (t)$ (see Eq. (13)). Its integral after weighing with $1/B_{\textrm{L}}$ gives the fraction of the contribution to the imposed limit of ${\dot{\varPsi }} / 4.$

A potential systematic effect associated with a *z* shift in the average orbit is a change in the acceptance of the upstream and downstream detectors. While we anticipate that identification of the *z* direction of the emitted positrons will remain unaffected, this aspect nevertheless requires a more comprehensive investigation. A more detailed discussion of detection-related systematic effects will be covered in an upcoming publication.

The limits shown in Fig. 8, result in specifications for the magnetic kicker used to rotate the momentum of injected muons into a stable orbit. To avoid systematic effects, it is necessary to limit the amplitudes below ${0.1}\,{\textrm{mT}}$ to ${1}\,{\textrm{mT}}$ in the frequency band between 30 kHz and 30 MHz, which will be measured using a laser-based Faraday rotation magnetometer, similar as described in [35]. Note that even in the case of a too large residual transient magnetic field from the kicker, its precise knowledge would allow us to correct for the systematic effect.

## Conclusions

In this study, we have presented analytical equations that describe in detail the precession arising from the AMM in the EM fields integral to the setup of the proposed muon EDM experiment at PSI. Our findings were verified using Geant4 Monte Carlo simulations that utilised realistic field maps generated by Ansys Maxwell.

We identified that the most relevant systematic effects stem from radial magnetic fields that vary with time and a non-zero longitudinal component of the electric field, i.e., parallel to the magnetic field. The effects of the longitudinal *E*-field can be largely mitigated by employing the CP-violating nature of the EDM by alternating periodically between CW and CCW particle motion in the storage ring. This can be achieved by switching the polarity of the currents generating the magnetic field, thus inverting the direction of the magnetic field, and correspondingly reversing the injection direction of the muons. The degree of this cancellation depends on the initial conditions of the experiment – the muon momentum distribution, the initial polarisation direction, the EM field setup and the overlap between the counter-rotating orbits.

We also provide a qualitative description of the geometric phase, accumulating as a result of spin oscillations in a non-uniform EM field. We found that systematic effects of substantial magnitude can only arise due to resonances between oscillations around two orthogonal axes, and only if the relative phase between the two oscillations is non-zero. One such effect may originate from the cyclotron motion in the electric field if the axis of this rotational-symmetric field is displaced and tilted with respect to the muon orbit’s central axis. Geometric phase accumulation due to oscillations with considerably different periods, such as cyclotron and betatron oscillations, has negligible impact.

The study presented here is a key contribution to the ongoing effort to search for the muon EDM at PSI. While specific calculations were presented for the initial phase of the experiment, the analytical derivations were kept sufficiently general so as to serve future upgrades aiming for higher sensitivity. The discussed systematic effects could also be relevant for other planned storage-ring EDM searches.

## Figures and Tables

**Fig. 9:**
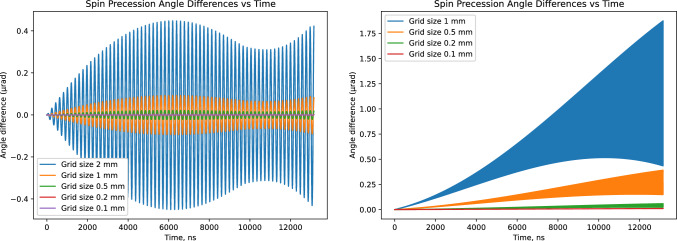
Time evolution of the spin angle difference as a function of the grid size for the *B*-field (left) or *E*-field (right). The expected signal should be in the order of ${21}\,{\upmu }\textrm{rad}/\upmu \textrm{s}$ and ${1.3}\,{\upmu }\textrm{rad}/\upmu \textrm{s}$ for the Phase I and Phase II experiment, respectively. *E*-field grid size of ${0.2}\,{\textrm{mm}}$ and *B*-field grid size of ${0.5}\,{\textrm{mm}}$ ensure sufficiently low deviations while still maintaining reasonable map size. Note that the curves appear like uniformly filled shapes due to very high frequency cyclotron oscillations

**Fig. 10:**
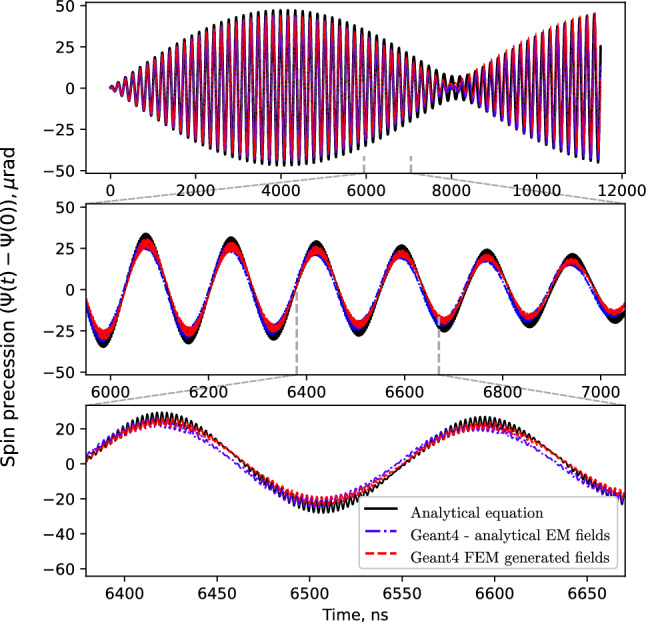
Comparison between the analytical Eq. (30) and the Geant4 spin tracking simulation. The initial parameters are arbitrarily set to $\vec {S}_0 = (-0.89, -0.46, 0.00),$ $\vec {p}_0 = (0.84, 0.55, 0.00) \times {26.8}\,\textrm{MeV}/c$ and $\vec {r}_0 = (-16.95, 22.10, 5.00)\,{\textrm{mm}}.$ The muon momentum is intentionally not set ideally to the ideal muon momentum for the frozen-spin field of $E_{\textrm{f}} = {280}\,{\mathrm{kV/m}}$ in order to highlight the $\omega _z$ oscillations of the spin. The three plots depict different timescales of the spin motion and show uncompensated $(g-2)$ precession (top), betatron oscillations (middle), and cyclotron oscillations (bottom)

**Fig. 11:**
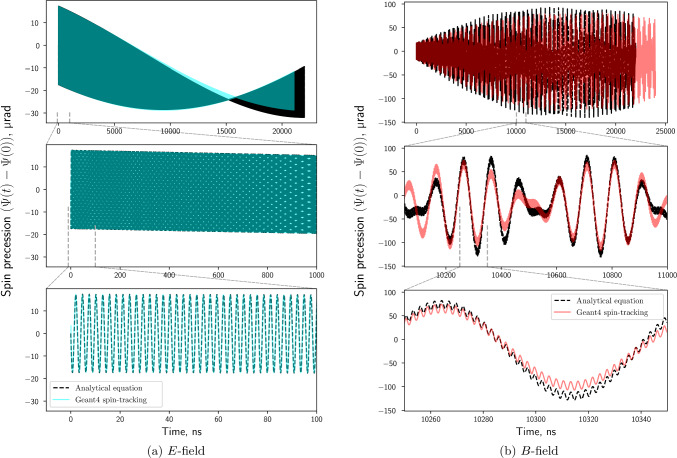
Accumulation of Berry’s phase due to oscillations of the spin around perpendicular axes. **a** Shows the case of equal oscillation frequencies (resonant oscillations) around the two axes caused by changing *E*-field in the muon reference frame. **b** Shows the Berry phase for the superposition of *B*-field oscillations with different frequencies (non-resonant oscillations) coupling to the *E*-field oscillations due to the cyclotron motion of the muon

**Fig. 12:**
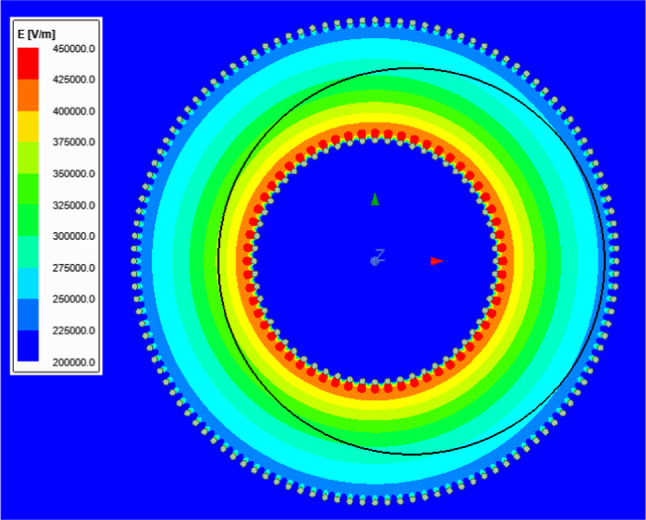
Radial electric field for ${20}\,{\textrm{mm}}$ inner charged electrode radius and ${40}\,{\textrm{mm}}$ outer grounded electrode radius when the electrodes are composed of ${1}\,{\textrm{mm}}$ diameter wires. The outer electrode has 120 and the inner 60 wires. The inner electrode high-voltage is ${-6200}\,{\textrm{kV}}.$ The black circle shows a ${6}\,{\textrm{mm}}$ displaced orbit

## Data Availability

This manuscript has no associated data or the data will not be deposited. [Authors’ comment: Data sharing not applicable to this article as no datasets were generated or analysed during the current study.]

## Appendix A: Verification using Geant4 spin tracking

### A.1 General considerations

To verify the analytical equations, a model of the experimental setup proposed was developed using the Geant4 Monte Carlo simulation toolkit. The EM fields of the experiment can be calculated analytically or interpolated from field maps. The field maps are generated by the ANSYS Maxwell3D FEM software. The simulation has three major EM field components:

1. The main solenoid magnetic field, with a constant value along *z*,  or a field map supplied by FEM simulations.
2. Radial electric field given by Eq. (24) with the option to add a constant or uniform component in the *z* direction or FEM simulated field map.
3. Weakly-focusing field modelled in ANSYS as a single circular coil with radius $R = {65}\,{\textrm{mm}}.$

Muons start with zero momentum in the *z* direction, since this is the initial condition for a stored muon. The simulation tracks the spin orientation in the reference frame of the muon and records it as a function of time. It can also track the direction with respect to a reference frame defined by the experimental setup, e.g. the solenoid magnet.

In terms of simulation requirements, the experimental setup of the muon EDM experiment is situated at the frontier between accelerator/storage ring physics and detector physics. The storage ring is fully contained within a single solenoid, and the beam focusing is performed by a circular coil. To maximise sensitivity, all positron detectors are located as close as possible to the stored muons. Thus, the use of the Geant4 toolkit is highly motivated as it provides an easy implementation of complex electromagnetic fields together with the detector geometries. However, its tracking (equation of motion integrator) is mainly developed for the purposes of single-pass systems. In the case of the muon EDM experiment, the muons travel a significant distance within the storage ring before decaying. The Phase I experiment muons perform 880 turns per muon lifetime, equivalent to about 180 m. Therefore, before proceeding with the verification of the analytical equations, the capabilities of the Geant4 simulation for accurate tracking and interpolation of EM field grids were tested.

### A.2 Verification of tracking and interpolation

To track the muons inside the storage ring volume, the Monte Carlo code requires the values of the EM fields at arbitrary positions in space, whereas all practically useful methods can only provide those on a grid. We have implemented a Catmull–Rom cubic spline [36] to interpolate the field at a point from a regular 3-dimensional field map. To benchmark the performance of the interpolator, we use an exact analytical solution of the *B*-field of a circular current loop [37] and calculate the field on a regular grid with step sizes from 0.1 to 2 mm. The numerical simulations were then launched with a ${28}\,{\textrm{MeV}/\textit{c}}$ muon starting from a fixed position, which was then tracked through the field once using its exact analytical expression or by interpolating it from the regular grid field maps. The difference in spin phase out of the orbit plane $\varPsi$ was calculated between the simulations using the interpolated field, or the exact solution. The results are shown in Fig. 9 (left, for the *B*-field, right, for the *E*-field cases). All grid sizes tested below ${2}\,{\textrm{mm}}$ result in an acceptable *B*-field approximation in relation to the expected Phase I or even Phase II target sensitivities, ${21}\,{\upmu }\textrm{rad}/{\upmu }\textrm{s}$ and ${1.3}\,{\upmu } \textrm{rad}/{\upmu }\textrm{s},$ respectively. The same study was done for the *E*-field interpolation by generating field maps with grid size from 0.1 to 1 mm from the exact analytical solution for coaxial cylindrical electrodes. As the spin of muons at that momentum is more sensitive to the *E*-field, the necessary grid spacing that ensures a good approximation is 0.2 mm or lower.

The sixth-order Dormand–Prince integration routine of Geant4 [9] was used for the tracking of muon position and spin throughout the simulations. To determine the optimal step size of the integrator, we performed tracking simulations with step sizes of fractions of $\sqrt{2}$ from 0.014 to 2.82 mm. The irrational numbers for the step size were chosen so as to avoid effects due to resonances between the integration step and field map grids. The smallest step size generates more than $10^4$ integration steps per rotation and was used as a reference. For all integration step sizes studied, the deviation of the spin phase $\varPsi$ from the reference trajectory was below ${0.1}\,{\textrm{nrad}/{\upmu }\textrm{s}}$ or four orders of magnitude below the signal at the statistical sensitivity of the Phase II experiment. However, a step size of $\sqrt{2}/2$ mm was chosen for future tracking, as it still results in a sufficiently short simulation time.

With this, the verification of the tracking and interpolation performance of the simulation is completed. We have proceeded to verify the ability of the FEM software to generate field maps that agree with the exact solutions for idealised coil and electrode shapes and the descriptive power of the derived analytical equations of spin motion.

### A.3 Verification of analytical equations

A model of the experimental setup was created in ANSYS Maxwell3D with a solenoid consisting of one main coil and two shimming coils on each side. The coil parameters (current density, radius, position, length, etc.) were obtained from a best fit of a measured field map of the superconducting solenoid that will be used in the Phase I muon EDM experiment. The circular coil that produces the weakly-focusing field is positioned in the centre of the solenoid. It produces a field that in the centre of the coil points in the opposite direction to that of the main solenoid, which serves as a potential well in which muons are stored. The simulation also includes a coaxial cylindrical electrode system $({20}\,{\textrm{mm}}$ inner and ${40}\,{\textrm{mm}}$ outer electrode radii) that provides the radial electric field for the frozen-spin condition. The FEM software is then used to calculate the field produced by the coils and electrodes and to output it on a regular grid.

A comparison between the analytical equations derived and the Geant4 spin tracking is shown in Fig. 10. The fields used were either provided by exact analytical solutions for the weakly-focusing coil and coaxial electrodes or field maps from numerical simulations. The initial coordinates of the muon at the moment of storage were arbitrarily chosen (values specified in the caption of Fig. 10) for illustration purposes. The electric field was set at such a value as to have imperfect cancellation of the $(g-2)$ precession. Both the inner and outer electrodes that generate the coaxial *E*-field are tilted around the same pivot with respect to the *z*-axis by $\delta ={0.01}^\circ$ to highlight the cyclotron oscillations. Simulations were performed for the ${1000}\,{\textrm{m}}$ track length, or about 7 muon lifetimes.

The comparison shows very good agreement between the analytical equations and the Geant4 spin tracking performed using field maps or exact solutions. The weakly-focusing field gradient $\partial _z B_\rho$ (needed to calculate the field index *n* in Eq. (30)) was calculated using an exact solution of a perfect circular current loop [37] at position $\rho = \rho _0$ and $z = 0.$ This description of the weakly-focusing field provides good estimates of the field strength and the betatron frequency. The difference between the exact and numerical approaches for field generation is negligible throughout the whole simulation time, demonstrating the good agreement between the two, also when using realistic field maps from a physical coil $({62}\,{\textrm{mm}}$ radius and ${5\,\textrm{mm} \times 5\,\textrm{mm}}$ square cross section) generated by the finite-element method.

### A.4 Verification of geometric-phase equations

For the verification of the equations derived for the calculation of the geometric phase accumulation, we consider two different cases: resonant and non-resonant oscillations.

#### A.4.1 Resonant oscillations

Two periodic oscillations along perpendicular axes with equal angular frequencies can occur due to the cyclotron motion of muons inside the *E*-field used for the frozen-spin technique. Consider the case described in Eq. (23), where there is an angle between the common axes of the electrodes and the solenoid magnetic field, and the centre of the muon orbit is displaced by $\vec {r}_0$ with respect to the centre of the electric field. Equation (27) shows that in this case, the net $(g-2)$ precession over a turn would be zero. The net precession due to the longitudinal *E*-field component seen in the muon reference frame would also be zero. However, the spin will cause small oscillations around the longitudinal and radial axes with the cyclotron angular frequency $\omega _{\textrm{c}}.$ A geometric phase will be observed if the displacement of the orbit $\vec {r}_0$ is such that both oscillations are out of phase.

To calculate the resulting geometric phase accumulation, we use Eq. (37), where $\omega = \omega _{\textrm{c}}.$ The amplitudes $A_z$ and $A_\rho$ are the maximum spin precession angular velocity resulting from the oscillations in the *z* and radial *E*-field components. Using the Thomas-BMT equation and Eq. (24) they can be approximated as

A.1 $$\begin{aligned} A_z&= -\frac{ea}{mc} \left[ 1 - \frac{1}{a(\gamma ^2 - 1)}\right] \beta _\theta \frac{\max (E_z) - \min (E_z)}{2} \text { and } \end{aligned}$$

A.2 $$\begin{aligned} A_\rho&= -\frac{ea}{mc} \left[ 1 - \frac{1}{a(\gamma ^2 - 1)}\right] \beta _\theta \frac{\max (E_\rho ) - \min (E_\rho )}{2}. \end{aligned}$$

The Geant4 simulation of the experimental setup was prepared with exaggerated parameters to highlight the phase accumulation effect to verify (37). The coaxial electrodes are tilted at ${15}\,{\textrm{mrad}}$ and the centre of the muon orbit is displaced by ${5}\,{\textrm{mm}}.$ The radial *E*-field is lower than that required for the frozen-spin condition to allow for a residual $(g-2)$ precession. The phase between the two oscillations is set to the worst-case scenario, i.e. $\pi /2.$ The comparison between the equations and the spin tracking simulation is shown in Fig. 11a. For the purpose of comparison, we assume classical parallel transport, since the simulation software is not capable of simulating quantum behaviour. In reality, phase accumulation will be half of the value obtained.

#### A.4.2 Non-resonant oscillations

To validate Eq. (36), the same simulation conditions as the previous comparison were used, but with an additional oscillating radial *B*-field. Spin oscillations caused by the *B*-field coupled with those in $E_z$ that are due to the ${8.7}\,{\textrm{mrad}}$ tilt of the electrode system. The source of the radial field in the simulation is the anti-Helmholtz pair of coils, which is a realistic scenario as in practice such radial oscillations can be induced by a residual *B*-field from the magnetic kick. The *B*-field in the comparison is generated by superposition of two currents of the form $I(t) = I_0 \sin (\omega t + b_0)$ with different amplitudes and initial phases and angular velocities at $\pm 5$% of the cyclotron angular velocity $({2.3}\,{\mathrm{rad/ns}}).$ The amplitudes of the two sinusoidal components are ${10}\,{\textrm{A}}$ and ${5}\,{\textrm{A}},$ which is more than an order of magnitude higher than the expected residual current after the magnetic kick. This is done to highlight the geometric phase effect, as otherwise it would be negligible. The effect is also enhanced by setting the tilt of the electrode system to such a large value, again for the purpose of illustrating the geometric phase effect.

The results of the comparison are presented in Fig. 11b. A very good agreement between theoretical prediction and spin-tracking simulations is observed on the microsecond scale $(g-2$ precession) and the nanosecond scale (cyclotron oscillations) over about 10 muon lifetimes. The overall behaviour of the geometric phase is captured by the analytical equations, though there are small differences between the predicted and observed beating patterns in case of the *B*-field coupling. The oscillation amplitude of the radial *B*-field generated from the magnetic kick was calculated at the $(x, y, z) = (\rho _0, 0, 0)$ position, whereas the muon experiences longitudinal and horizontal betatron oscillations, thus resulting in a slightly different *B*-field. Despite this approximation, the agreement between the simple calculation and the detailed spin tracking is satisfactory. Other sources of difference between the analytical and simulation approaches could come from the discretisation of the *B*-field used in the numerical simulation, which introduces additional high-frequency noise, and from the calculation of the cyclotron frequency, which is proportional to be *B*-field. In the analytical calculation we have used the ${3}\,{\textrm{T}}$ main solenoid field, which does not include the effects of the weakly-focusing coil or the variable field due to the kick. Differences could also be due to the motion of the muons in space, since the generated field is the sum of two sines, but the field in the rest frame of the particle would have higher- and lower-frequency terms.

## Appendix B: Striped electrode system

The limits shown in Sect. 3.3 place stringent constraints on the alignment and shape of the electrode system. Although the simplest way to achieve a radial *E*-field is to employ a coaxial cylindrical structure, these limits could be achieved more easily with a more complex setup, e.g. inner and outer electrode geometries that are composed of individual wires instead of a uniform cylindrical foil.

Such a setup would introduce radial non-uniformity along the circular muon orbit, which could lead to the accumulation of a geometric phase. However, as seen in Fig. 3, the limits on the electric field uniformity are quite relaxed for high-frequency oscillations. For example, separating the electrodes into 100 longitudinal strands would create radial field non-uniformity. If there is a tilt of the electrode system, that would translate to an $E_z$ non-uniformity as well. However, the frequency of the *E*-field oscillations due to this non-uniformity will be at about ${40}\,{\textrm{GHz}}.$ At such high frequency oscillations of the radial component on the level of even 50% would produce a negligible geometrical phase accumulation. Note, however, that the geometrical phase calculations assume adiabatic processes and such extreme conditions might violate that assumption. FEM simulations of the electric field generated by an electrode segmented into segmented into 60 parts, each consisting of a ${1}\,{\textrm{mm}}$ diameter wire and a grounded electrode segmented into 120 wires show that the amplitude of the high-frequency field oscillations are less than 1% even for a displacement of ${6}\,{\textrm{mm}}$ of the centre of the muon orbit. The transverse cross section of the electric field and the displaced orbit are shown in Fig. 12. The geometrical phase effect of such an electrode structure is shown by the red ellipsoid in Fig. 4.

The main reason to prefer a wired electrode setup is that in practice it would be easier to construct uniform electrodes compared to using cylindrical foils. One can devise a setup where the wires are connected to piezoelectric actuators that allow for very fine control of the position. The actual position of the wire can then be measured with sub-micrometer precision using optical or capacitive distance sensors. The produced electric field can then be precisely simulated using finite-element methods.

Another advantage of a striped system is the reduced material budget, which would increase the path length of decay positrons and reduce their scattering. Compared to a cylindrical foil, it would also lead to significantly less eddy currents from the magnetic kick.

## Footnotes

1 Note that an equivalent effect exists also in classical mechanics [26].

2 Here we use $F=1/4$ to allow for up to 16 independent systematic effects, each at a quarter of the statistical sensitivity. This ensures that the total systematic uncertainty remains on par with statistical uncertainty, given that uncertainties are combined quadratically in the final analysis. Such a choice is naturally arbitrary and depends on the requirements of the particular experiment.

3 In the context of Penning traps the horizontal betatron frequency is commonly referred to as the reduced cyclotron frequency.

## Code availability

My manuscript has no associated code/software. [Author’s comment: Figures 9, 10 and 11 were generated using a Geant4 code with is available from the authors upon request.]
